# A genetic mouse model of lean-NAFLD unveils sexual dimorphism in the liver-heart axis

**DOI:** 10.1038/s42003-024-06035-6

**Published:** 2024-03-22

**Authors:** Charlotte Burelle, Valentin Clapatiuc, Sonia Deschênes, Alexanne Cuillerier, Marine De Loof, Marie-Ève Higgins, Hugues Boël, Caroline Daneault, Billie Chouinard, Marie-Élaine Clavet, Nolwenn Tessier, Isabelle Croteau, Geneviève Chabot, Catherine Martel, Martin G. Sirois, Sylvie Lesage, Yan Burelle, Matthieu Ruiz

**Affiliations:** 1 Department of Medicine, Université de Montréal, Montreal, QC Canada; 2 Research Center, Montreal Heart Institute, Montreal, QC Canada; 3 Faculty of Health Sciences and Medicine, University of Ottawa, Ottawa, OC Canada; 4 Research Center, Maisonneuve-Rosemont Hospital, Montreal, QC Canada; 5 Department of Physiology and Pharmacology, Université de Montréal, Montreal, QC Canada; 6 Department of Nutrition, Université de Montréal, Montreal, QC Canada

**Keywords:** Metabolism, Metabolic disorders

## Abstract

Lean patients with NAFLD may develop cardiac complications independently of pre-existent metabolic disruptions and comorbidities. To address the underlying mechanisms independent of the development of obesity, we used a murine model of hepatic mitochondrial deficiency. The liver-heart axis was studied as these mice develop microvesicular steatosis without obesity. Our results unveil a sex-dependent phenotypic remodeling beyond liver damage. Males, more than females, show fasting hypoglycemia and increased insulin sensitivity. They exhibit diastolic dysfunction, remodeling of the circulating lipoproteins and cardiac lipidome. Conversely, females do not manifest cardiac dysfunction but exhibit cardiometabolic impairments supported by impaired mitochondrial integrity and β-oxidation, remodeling of circulating lipoproteins and intracardiac accumulation of deleterious triglycerides. This study underscores metabolic defects in the liver resulting in significant sex-dependent cardiac abnormalities independent of obesity. This experimental model may prove useful to better understand the sex-related variability, notably in the heart, involved in the progression of lean-NAFLD.

## Abstract (web summary)

A genetic mouse model of lean-NAFLD reveals that male exhibit a more severe hepatic phenotype with increased macrophage infiltration, FGF21 concentration and sustained hypoglycemia associated with cardiac diastolic dysfunction.

## Introduction

Non-alcoholic fatty liver disease (NAFLD) affects ~25% of the world population and is associated with metabolic comorbidities, including obesity, type 2 diabetes, hyperlipidemia, and hypertension^1^. Currently, the most frequent cause of death in NAFLD is cardiovascular diseases (CVDs). Although regularly associated with overweight or obesity, NAFLD is increasingly being identified in lean individuals. In fact, a 2020 meta-analysis estimated that among the NAFLD population, the overall prevalence of non-obese NAFLD is just over 40%, with ~1 in 5 patients being lean (BMI < 25 kg/m^2^ ^2^). Furthermore, it has been repeatedly suggested that NAFLD in overweight and obese individuals and NAFLD in lean people represent distinct pathophysiological entities (reviewed in ref. ^3^). More specifically, there is a marked overlap between NAFLD in lean people and metabolic dysfunction-associated fatty liver disease (MAFLD) when considering the overall clinical profile and metabolic health^4^.

Data on the long-term cardiovascular outcome of NAFLD in lean people are limited. Yet, they show that lean individuals with NAFLD have a lower prevalence of metabolic comorbidities (e.g., diabetes, hypertension, hypertriglyceridemia, obesity, metabolic syndrome), but higher liver fibrosis scores and elevated cardiovascular morbidity and all-cause mortality in advanced stages compared to obese individuals with NAFLD (reviewed in ref. ^5^). Considering the greater cardiovascular risk associated with NAFLD in lean people, it is crucial to better understand the mechanisms leading to the development of cardiac abnormalities in this population.

In the obese population, NAFLD is accompanied by a constellation of systemic alterations and comorbidities that collectively contribute to the development of cardiovascular complications (reviewed in ref. ^6^). This includes, but is not limited to, adipose tissue dysfunction^7^, ectopic deposition of fatty acids in peripheral tissues (e.g., skeletal muscles, heart^8^), insulin resistance as well as hyperglycemia and its adverse cellular consequences (e.g., formation of reactive oxygen species and pro-inflammatory processes^9^). Nevertheless, a significant proportion of lean patients with NAFLD do not present most of these systemic alterations and comorbidities, which suggests that the liver pathology itself plays a predominant role in the pathogenesis of cardiac complications. However, it is challenging to experimentally dissociate liver pathology from systemic disturbances and comorbidities.

Abnormal lipid accumulation in the liver is pathognomonic for NAFLD. As major regulator of lipid metabolism, it is well known that mitochondrial dysfunction induced by lipotoxicity is closely associated with the progression of the obese NAFLD phenotype, and its extra-hepatic manifestations including CVDs (reviewed in ref. ^10^). Noteworthy, NAFLD has many systemic consequences and its relationship with CVD is complex. NAFLD is considered to be a significant, and most importantly, an independent risk factor for CVD^10^. The progression of NAFLD is associated with changes in cardiac morphology and impaired diastolic function^11,12^. To study such cardiac abnormalities of a NAFLD in the absence of obesity and independently of any pre-existent metabolic disruptions and comorbidities, we took advantage of our hepatic *Lrpprc* knockout (KO) mouse model (H-LRPPRC) characterized by a mitochondrial dysfunction consequently to a defect in the assembly of respiratory chain complex IV^13–15^.

This model has been previously depicted by its mitochondrial hepatopathy in the absence of obesity, recapitulating to some extent the circulating lipidomic signature described in NAFLD (e.g., higher levels of hepatic triglycerides (TGs) and long-chain acylcarnitines (LCACs), as well as changes in plasma glycerophospholipids^14,16^. The aim of the present study was, therefore, to characterize the cardiac abnormalities of NAFLD without obesity. Considering the notable impact of sex in the initiation and progression of this disease^17^, we also assessed the sex impact. Our results reveal that loss of hepatic LRPPRC triggers a multi-component phenotypic remodeling ranging beyond liver damage, with notable disturbances in glucose metabolism and metabolic and functional cardiac impairments, which are shown to be sex-dependent, highlighting that metabolic and contractile abnormalities are not necessarily coupled in this model. This study gives new insight into the crucial involvement of mitochondria in the pathophysiology of NAFLD without obesity and its impact on CVDs development and progression.

## Results

### Loss of hepatic LRPPRC does not lead to obesity

**Fig. 1:**
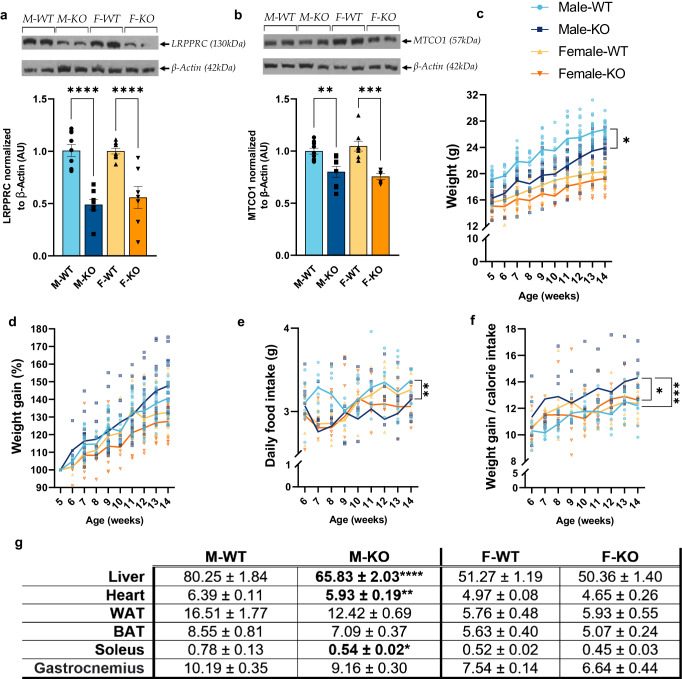
General phenotype in normal and liver-specific LRPPRC deficient mice. Immunoblots of **a** LRPPRC, and **b** MTCO1 content in the liver (*n* = 10). β-Actin was used as loading control. You can find the uncropped/unedited immunoblots for each protein content and their aligned size markers in Supplementary Fig. 4. **c** Mice weight and their **d** weight gain (in %) were measured and calculated once a week. **e** Daily food intake, and **f** calorie assimilation represented by weight gain on daily caloric intake were also calculated once a week. **g** At 14 weeks of age, organ mass normalized to tibia length was calculated (mg/mm). The table represents the average mass for each group and organ ± SEM. Statistics: difference between WT and KO mice was assessed with a two-way ANOVA test for **a**, **b**, **g**. One-way ANOVA test was performed for panels **c**–**f** thus considering the mean value for each group for the parameter analyzed in each panel. Each statistical test was followed by Šidák multiple comparison post hoc analysis. *p < 0.05, **p < 0.01, ***p < 0.001, ****p < 0.0001.

*Lrpprc* knockout (KO) model was validated by the 60% hepatic decrease in LRPPRC protein content similarly in both KO male and female mice (Fig. 1a). As expected, this hepatic loss induced a significant and sex-independent decrease of the respiratory chain complex IV subunit 1 protein content (MTCO1; Fig. 1b). Noteworthy, our results show the presence of residual amounts of LRPPRC which are likely attributable to liver regeneration^14^ and are still consistent with our previous study^16^.

Throughout the entire follow-up, homozygous KO mice had reduced body weight compared to their respective controls (Fig. 1c). Despite a lower weight also observed in KO females, significance was only reached in KO males when considering the mean value of weight for each group (Fig. 1c). However, KO males and females had similar weight gain compared to their control counterparts (Fig. 1d). Of note, the weight of WT mice was subject to sexual dimorphism, a maintained pattern in KO mice. Consistent with these results, the absence of increased lipogenic tissue mass (white adipose tissue, WAT; Fig. 1g) corroborated the absence of obesity in KO mice.

To rule out the effect of diet and food intake on the observed phenotype, the daily food intake (Fig. 1e) of mice and their ability to assimilate food (weight gain/calorie intake; Fig. 1f) were calculated. As shown in Fig. 1e, a significant decrease in daily food intake was observed only in KO males, when considering the mean value of daily food intake for each group. Furthermore, only KO females had a similar weight gain/food intake profile compared to their controls suggesting that, despite a somewhat different daily intake, they would not have a problem assimilating calories (Fig. 1f). A difference was however more evident in KO males than in KO females, as they displayed a significant increase in weight gain/calorie intake curve compared to their controls (Fig. 1f). Together, these results indicate that the phenotype of H-*Lrpprc* deficient mice, especially in female, cannot be explained by a difference in food or caloric intake.

To better understand the nutritional and health status of the *Lrpprc* KO mice, anthropometric parameters at 14 weeks were assessed. Normalized to the tibia’s length, liver mass of KO males was 18% smaller than their controls in a significant manner (*p* value < 0.0001) (Fig. 1g). This significant decrease was, however, lost when normalized to body mass (Supplementary Table 2) indicating that even though the loss of LRPPRC seems to affect liver mass, this decline was perhaps not explained by liver atrophy. No changes were observed in KO females. Figure 1g also shows a similar pattern found in the heart and soleus. Indeed, heart and soleus masses were significantly decreased by 11% and 18%, respectively in KO males compared to WT. However, this significant difference was only present when normalizing to tibia length, suggesting once again that this loss of mass is probably not related to muscle atrophy but rather to delayed growth (Supplementary Table 2). This result was consistent with our previous study^14^ as well as with LSFC patients harboring the LRPPRC mutation A354V where a growth delay is well documented^18–20^. Yet again, no difference was noted in KO females. Interestingly, the mass of the gastrocnemius, a glycolytic muscle, was unchanged in both sex (Fig. 1g). Finally, the mass of lipogenic tissue—white (WAT), and brown (BAT) adipose tissue—were unchanged regardless of genotype (Fig. 1g). It should be noted that the absence of an increased WAT mass was consistent with body weight follow-up, demonstrating the absence of obesity in this model. Altogether, these results corroborated those previously obtained at 5 weeks of age^14^, and confirmed the lack of obesity in our model, which can thus be characterized as lean.

### Loss of hepatic LRPPRC results in deleterious morphological liver remodeling regardless of sex as well as sex-specific inflammatory profile

**Fig. 2:**
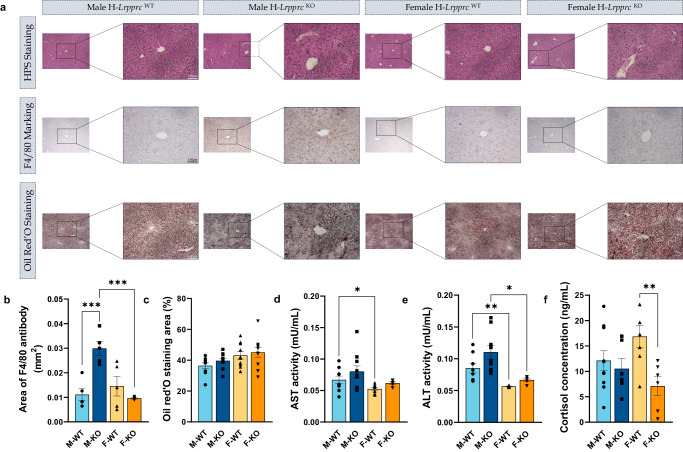
Liver phenotype in normal and liver-specific LRPPRC deficient mice. **a** Representative images of HPS staining, F4/80 antibody marking and Oil Red’O staining  in livers from WT and KO mice. The small images of each group are ×10 magnification, and the zoomed images are ×20 magnification. Scale bars are shown only for the first ×20 magnification of each group and represent 100 μm. **b** F4/80 antibody immunohistological marking area was quantified in mm^2^ and **c** Oil Red’O staining area was quantified in percentage in histological slides from *Lrpprc* WT and KO livers (*n* = 10). The activity of **d** aspartate and **e** alanine aminotransferases was measured (*n* = 5–10). **f** Quantified circulating cortisol levels (*n* = 6–10). Statistics: two-way ANOVA followed by Šidák multiple comparison post hoc analysis for normally distributed data in **b**, **c**; Kruskal–Wallis ANOVA test followed by Dunn’s uncorrected test for non-normally distributed data (**d**–**f**). *p < 0.05, **p < 0.01, ***p < 0.001.

To assess and validate the liver phenotype, a series of histological and molecular analyses were performed. As described in the Histology section, to assess liver structure in all phenotypes, we first used HPS staining, which stains protein content as well as DNA and cytoplasm in order to visualize hepatic structures such as bile ductules, arterioles, sinusoids, hepatocytes, and portal veinules. Following HPS staining and qualitative observations, livers lacked structure and organization in KO males and females in a similar fashion compared to their controls (Fig. 2a). More specifically, disorganization was noted in KO mice through less structurally defined portal veinules, arterioles, and sinusoids and a more disorganized and less condensed volume of hepatocytes. In addition, portal veinules were visually more dilated in KO mice (Fig. 2a), whereas no microvesicular steatosis was noted following Oil Red’O staining (Fig. 2a, c) in contrast with what was previously seen at 5 weeks of age^14^. Nonetheless, when quantifying the area occupied by the F4/80 antibody, a substantial difference emerged (Fig. 2b). The area percentage was markedly greater in KO males in comparison to WT males (Fig. 2b). This outcome strongly implies an increased presence of macrophage infiltration, suggesting heightened engagement of immune cells in the livers of KO males. Consequently, it prompts the hypothesis that KO males undergo a more progressed state of liver disease, involving a greater abundance of immune cells than observed in KO females. To assess hepatocellular injury, alanine, and aspartate aminotransferases (ALT and AST) activities were measured. AST, produced in hepatocytes as well as in muscle and heart cells, was not significantly different in both males and females except for the WT groups (Fig. 2d). ALT, produced mainly, but not exclusively, in hepatocytes, is, however, showing a trend towards a 1.29-fold increase in KO males without reaching significance (Fig. 2e). KO male and female mice were however significantly different (2-fold higher in males).

**Fig. 3:**
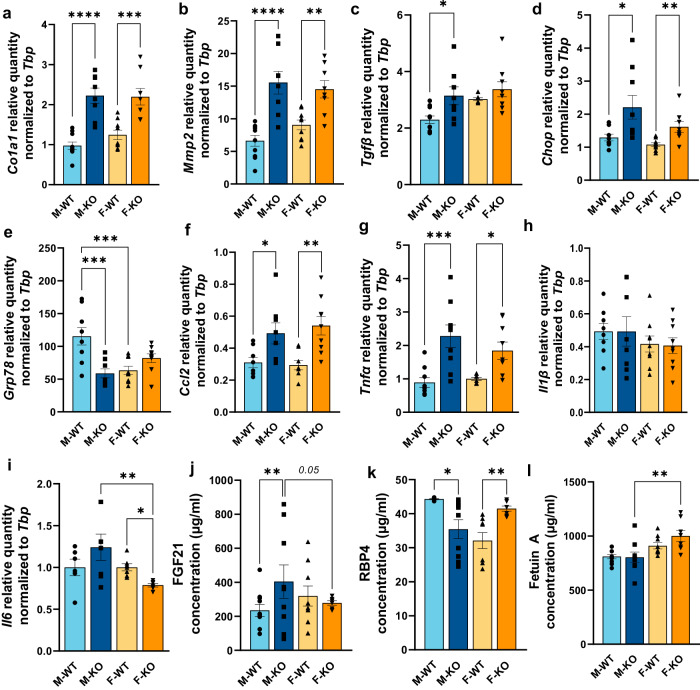
RT-qPCR analyses in liver and ELISA analyses in plasma of several hepatic markers in normal and liver-specific LRPPRC deficient mice. mRNA levels quantified by RT-qPCR in livers from WT and KO mice for **a** *Col1a1* for fibrosis, **b** *Mmp2* and **c** *Tgfβ* for matrix remodeling, **d** *Chop* and **e** *Grp78* for ER stress **f** *Ccl2*, **g** *Tnfα*, **h** *Il1β* and **i** *Il6* for inflammation. Circulating hepatokines were quantified by ELISA: **j** FGF21, **k** RBP4, and **l** Fetuin A. Statistics: two-way ANOVA test followed by Šidák multiple comparison post hoc analysis for normally distributed data in **a**–**c**, **f**, **g**, and **j**–**l**; Kruskal–Wallis ANOVA test followed by Dunn’s uncorrected test for non-normally distributed data in **d**, **e**, **h**, **i**. *p < 0.05, **p < 0.01, ***p < 0.001, ****p < 0.0001.

To further identify the extent of liver damage, several hepatic markers were targeted. mRNA levels of fibrosis marker, *Col1a1*, and matrix remodeling marker, *Mmp2*, were first quantified. *Col1a1* expression was significantly increased by 2.28- and 1.77-fold in KO males and females, respectively (Fig. 3a). Consistent with this result, *Mmp2* expression was also increased by 2.35-fold in KO males and by 1.60-fold in KO females (Fig. 3b) suggesting liver matrix remodeling with the presence of fibrosis which, however, was not histologically confirmed. In contrast, *Tgfβ* was only significantly increased in KO males (Fig. 3c). The presence of liver damage and remodeling led us to evaluate some markers of endoplasmic reticulum (ER) stress. As such, *Chop* expression was significantly increased in KO males (1.71-fold) as well as in KO females, although in a less pronounced fashion (1.49-fold) (Fig. 3d). However, the expression of *Grp78* was significantly decreased by 0.51-fold in KO males, but slightly increased in their female counterparts without reaching significance (1.30-fold, *p* = 0.2598; Fig. 3e). This increase in markers of ER stress has been observed and associated with fibrosis and the exacerbation of liver diseases in mice^21^. The earlier demonstrated outcome of marked F4/80 staining in KO males drew our focus towards other indicators associated with inflammation. As shown in Fig. 3f, *Ccl2* expression was once again significantly and similarly increased in both KO males and females (1.59- and 1.84-fold, respectively). There was also a notable upsurge in *Tnfα* mRNA levels in both sexes (Fig. 3g), while no distinctions were detected in *Il1β* mRNA expression (Fig. 3h). Finally, mRNA levels of *Il6* exhibited significant disparities between KO males and females (Fig. 3i). Indeed, KO males exhibit higher levels of *Il6* mRNA, despite not reaching statistical significance when compared to M-WT whereas KO females show a significant decrease (Fig. 3i). Noteworthy, in an effort to broaden our findings and encompass a more comprehensive inflammation profile, we performed a comprehensive survey of plasma cytokines using a multiplex strategy. Unfortunately, most of the acquired signals fell below the detection threshold. The sole signals having surpassed this detection threshold are illustrated in supplementary Fig. 1 where none showed a significant difference except for IL-10 concentration with a twofold increase in KO males compared to KO females (Supplementary Fig. 1b). Interestingly, the Tnfα plasma concentration profile (Supplementary Fig. 1a) reflects a similar profile to the hepatic *Tnfα* expression profile (Fig. 3g). Overall, these results point out to a remodeling of the liver matrix with signs of fibrosis and inflammation that are independent of sex for most of the analyzed parameters.

To better delineate the systemic consequences of this NAFLD in lean mice in a sex-dependent manner, we performed ELISA assays for circulating hepatokines, namely, Fibroblast Growth Factor 21 (FGF21), Retinol Binding Protein 4 (RBP4), and Fetuin A also referred to as alpha2-HS-glycoprotein. As such, we observed a significant increase in circulating FGF21 levels in KO males while no differences were observed in females (Fig. 3j). Moreover, ELISA assay for RBP4 concentration revealed a significant decrease in KO males while showing a significant increase for KO females (Fig. 3k). The assay for Fetuin A revealed only a significant difference between KO males and females with no significant difference when compared to their respective control group (Fig. 3l). Since plasma levels of FGF21 are thought to reflect the severity of hepatic steatosis, this result suggests that KO male mice suffer from a more severe state of liver damage. This result, combined with the analysis of mRNA levels of different markers and particularly the increased staining of the F4/80 molecule in livers, seems to point towards a greater disease progression in KO males. It should be noted that a significant decrease in circulating cortisol was only observed in KO females (0.42-fold, Fig. 2f), which has previously been reported in patients with more advanced stages of NAFLD^22^.

**Fig. 4:**
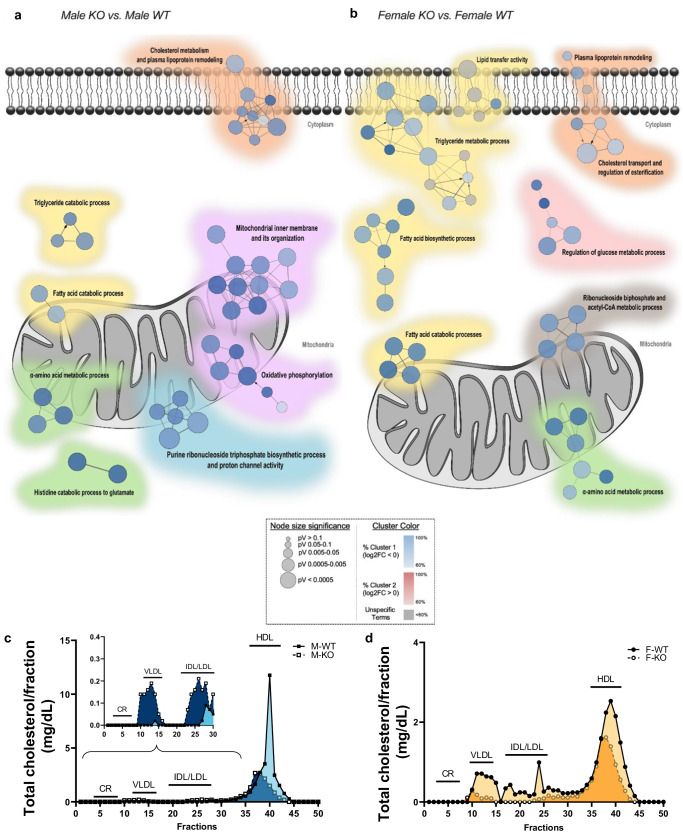
RNA-sequencing analysis in the liver and total cholesterol level in plasma lipoprotein fraction of normal and liver-specific LRPPRC deficient mice. The liver transcriptomic signature of KO **a** male and **b** female mice compared to their respective control (*n* = 10). Transcripts were classified as down- (blue, cluster #1) or upregulated (red, cluster #2) based on their log2 fold-change. For each comparison, the Cytoscape ClueGO plugin was used to cluster transcripts significantly enriched following enrichment analyses of GO terms (Molecular Function, Cellular Component, and Biological Process) and pathways (KEGG database). The size of the nodes reflects the statistical significance of the terms (pV). The node color shows the proportion of transcripts from each cluster that are associated with the term. Difference between WT and KO mice was assessed with a classical Fisher’s exact test following by a Bonferroni step-down multiple comparison post hoc analysis. Only metabolic pathways with a pV ≤ 0.05 are shown here. Images from **a**, **b** were created from RNA-sequencing data generated by Palidwor G., Ottawa Bioinfor- matics Core Facility (OBCF). Plasma from mice at 14 weeks of age was pooled and subjected to gel filtration by FPLC. Total cholesterol in each lipoprotein fraction (total of 60) was measured in **c** males (*n* = 4), and **d** females (*n* = 4) mice.

To dig deeper into the hepatic signature of our *Lrpprc-null* mice and whether there could be a transcriptomic sexual dimorphism, RNA-sequencing analysis was performed. As shown in Fig. 4, the liver transcriptome signature of KO mice, in addition to being different from WT mice, was significantly impacted by sex. Males are especially characterized by decreased transcript markers of mitochondrial inner membrane organization and impaired oxidative phosphorylation (Fig. 4a). Consistently, purine ribonucleoside triphosphate biosynthetic process was also decreased. As for KO females, they showed a decrease of transcript markers for (i) the regulation of glucose metabolism (ii) the metabolism of ribonucleoside biphosphate and Acetyl-CoA, and (iii) the processes for fatty acid transfer and biosynthesis as well as triglycerides metabolism (Fig. 4b). Some similarities were however found in both sexes. As such, both KO males and females showed a decrease in transcripts involved in the metabolism of (i) ɑ-amino acids, (ii) plasma lipoproteins and cholesterol, and (iii) fatty acids and triglycerides catabolism. It should be noted that the analyses revealed only 8 transcripts (0.08% of all transcripts) expressed in a different and significant way between males and KO females (as opposed to 165 transcripts between WT (1.65% of all transcripts)) (Supplementary Table 3). Altogether, these results suggest a sexual dimorphism in the hepatic expression of many transcripts. While KO males showed a decrease in transcripts involved in mitochondrial organization and its phosphorylative capacity, KO females had a more pronounced decrease in transcripts involved in lipid metabolism.

Since the transcriptional profile of KO mice showed hepatic alteration of transcripts associated with cholesterol and plasma lipoprotein metabolism, this prompted us to measure total cholesterol in plasma lipoprotein fractions (Fig. 4c, d). Compared to their respective controls, KO males exhibited higher concentrations of VLDL-C and IDL/LDL-C (Fig. 4c), whereas KO females showed decreased levels of the same lipoproteins (Fig. 4d). As the endogenous lipoprotein pathways begin in the liver with the formation of VLDL, it is worth noting that the liver transcriptome of both KO males and females showed a decrease in several apolipoprotein-associated transcripts synthetized in the liver (e.g., *Apoa1/2*, *Apob*, *Apoc1/2/3*, *Apoe*; Supplementary Data 2–3). Interestingly, *Apoc1/3* and *Apoa5* transcripts—synthetized exclusively in the liver—were only decreased in KO males (Supplementary Data 3) suggesting a potential link between increased circulating VLDL-C and IDL/LDL-C^23^. However, a decrease in *Pcsk9* transcripts, which regulate the rate of degradation of LDL receptors, was observed in female KO (Supplementary Data 2) hinting a potential association with decreased circulating LDL-C. Finally, HDL concentration was decreased in both KO males and females (Fig. 4c, d). Since ApoA-I and -II are the core structural proteins of HDL, it is interesting to mention that the decrease in their transcriptional levels may have impacted the HDL profile in KO mice. No changes were observed in chylomicron remnants (CR) concentrations (Fig. 4c, d) suggesting that hepatic loss of LRPPRC does not impact lipid absorption capacity. Altogether, these results suggest again a sexual dimorphism in plasma lipoprotein levels with KO males exhibiting increased levels of VL/LDLs and decreased levels of HDL whereas KO females exhibit a general decrease in all lipoproteins.

### Loss of hepatic LRPPRC induces disruptions in glucose metabolism in both sexes, predominantly in males

**Fig. 5:**
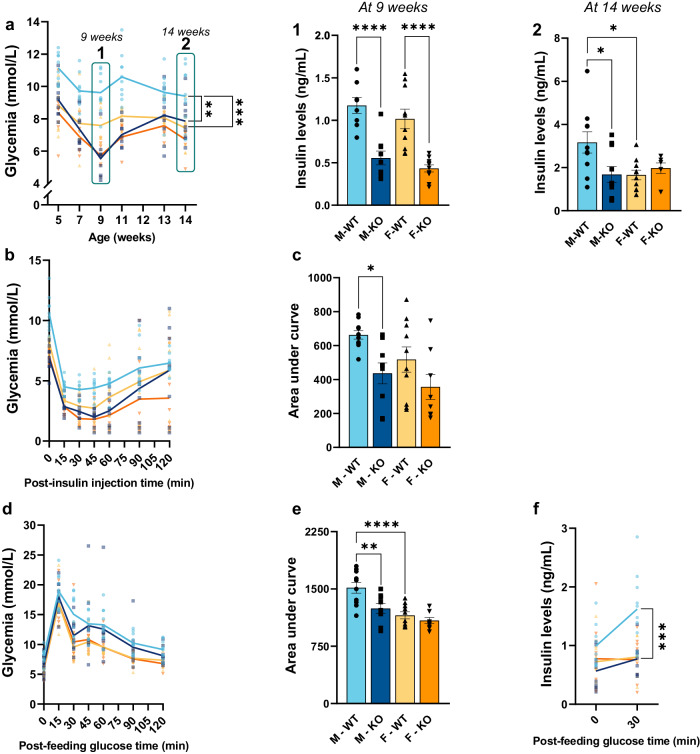
Glucose metabolism in normal and liver-specific LRPPRC deficient mice. **a** Blood glucose was monitored for 14 weeks, and insulinemia was quantified at 9 and 14 weeks (**1** & **2** respectively). ITT was performed at 11 weeks after a 5-hour fast to assess insulin sensitivity with the blood glucose curves (**b**), and the areas under the curves (**c**). OGTT was then performed at 12 weeks after a 16-hour fast to estimate the ability to secrete insulin, in response to a bolus of glucose, with the blood glucose curves (**d**) and the areas under the curves (**e**). Insulinemia was measured before the test (T0), and 30 minutes after glucose administration (T30) (**f**). Statistics: differences between WT and KO mice were assessed with a one-way ANOVA test for Figs. represented as curves **a**, **b** & **d** and with a two-way ANOVA for figures **a1** & **2**, **c**, **e** & **f** followed by Šidák multiple comparison correction. *p < 0.05, **p < 0.01, ***p < 0.001, ****p < 0.0001.

Since, in this mouse model, the loss of LRPPRC is specific to the liver, a key organ for gluconeogenesis, glucose metabolism is thus of interest for further analysis. A 14 weeks blood glucose follow-up showed that KO males displayed a hypoglycemic profile after a 5h-fast compared to their respective controls (Fig. 5a). Insulin deficiency is also observed (0.42-fold in males, and 0.46-fold in females) at 9 weeks of age (Fig. 5a1). At 14 weeks, this decrease remained evident in KO males only (0.57-fold decrease) whereas no changes were apparent in KO females (Fig. 5a2).

To gain further insight into the difference between *Lrpprc* WT and KO mice, an ITT was performed after a 5-h fast to assess insulin sensitivity. As shown in Fig. 5c, both KO males and females demonstrated a lower area under the curve (AUC) for glucose levels compared to their controls (0.66- and 0.69-fold, respectively). However, significance is only reached in KO males.

We next assessed the ability to secrete insulin in response to a bolus of glucose by performing an OGTT following a 16-h fast. KO males demonstrated a sudden decrease in glucose levels at 30 minutes post-gavage (Fig. 5d), resulting in a decrease of 0.84-fold AUC compared to their controls (Fig. 5e). Insulin levels between 0- and 30-minutes post-gavage were not significantly increased in KO males as after 30 minutes they had lower insulin levels as seen by a 0.48-fold decrease compared to WT males (Fig. 5f). Consistent with prior results, no significant changes were found in KO females in response to this glucose challenge. Overall, KO males were more affected than their female counterparts as they displayed pronounced fasting hypoglycemia and increased and persistent insulin sensitivity over a 14-week follow-up. This was consistent with the ITT and OGTT results where KO males exhibited insulin hypersensitivity associated with enhanced peripheral glucose reuptake.

In order to identify potential underlying mechanisms, we targeted key enzymes of gluconeogenesis, and glycogenesis (Supplementary Fig. 2). It is worth noting that even though no significant changes were observed, PEPCK content—the rate-limiting enzyme of neoglucogenesis—was modestly, without reaching significance, decreased in the liver of KO males whereas no changes were found in KO females. Interestingly, this pattern was reversed in glycogen synthase (GS) content, a key enzyme of glycogenesis where, compared to their respective controls, a 0.67-fold decrease was observed in KO females while KO males did not exhibit any significant changes despite still showing a significant difference with KO females.

### Loss of hepatic LRPPRC leads to a remodeling of the cardiac lipidome

**Fig. 6:**
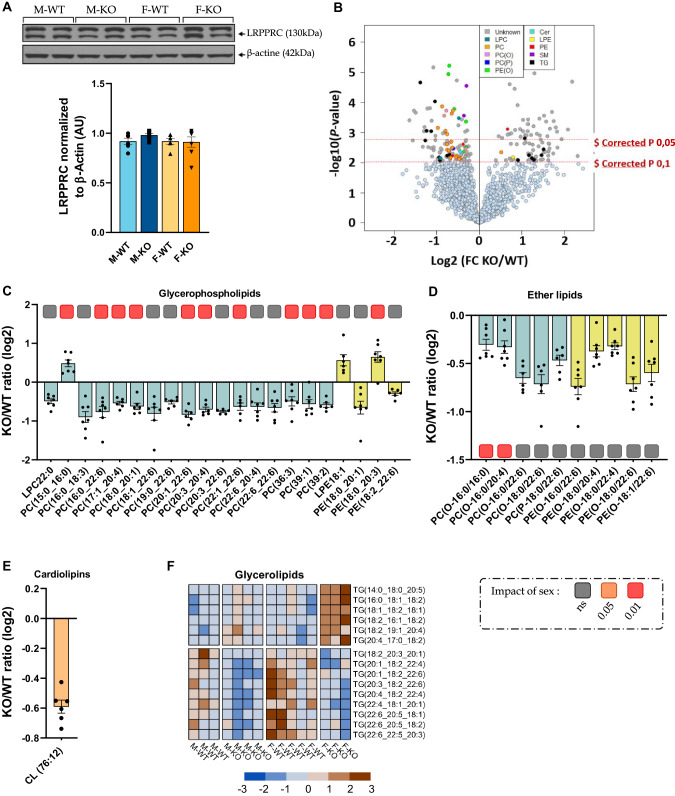
Untargeted lipidomic in hearts of normal and liver-specific LRPPRC deficient mice. **a** Immunoblot of LRPPRC content in the heart (*n* = 10) normalized to β-Actin protein. You can find the uncropped/unedited immunoblots for cardiac LRPPRC protein content and their aligned size markers in Supplementary Fig. 5. **b** Volcano plot from LC-QTOF-based untargeted lipidomic of hearts from Lrpprc KO and control mice (*n* = 7, 8/group) depicting the 2214 MS features obtained following MS data processing. The *x* axis corresponds to fold changes (FCs) of MS signal intensity values for all of these features in Lrpprc KO compared to control mice (log2). The *y* axis corresponds to the *p* values (−log10). Using a corrected p value (corrected P) threshold of 0.1 and 0.05 (horizontal red dotted lines), 162 MS features significantly discriminated Lrpprc KO from controls mice. See also Supplementary Data 5 for the list of lipids identified by MS/MS with FCs and *p* values (paired Student’s *t* test followed by Benjamini–Hochberg correction). **c**–**e** Selected lipids significantly ($p-corr<0.1) discriminating KO from controls mice and identified by MS/MS by LC-QTOF. Each dot represents a log2-transformed KO/WT signal intensity ratio (*n* = 7, 8) for the indicated lipid (sub)classes with their acyl side chain(s)—**c** glycerophospholipids: lysophosphatidylcholine (LPC) and phosphatidylcholine (PC) in teal, lysophosphatidylethanolamine (LPE) and phosphatidylethanolamine (PE) in yellow/green, **d** ether lipids, **e** cardiolipin (CL) 76:12. **f** Heatmap of glycerolipids (triacylglycerol; TG). The underscore symbol “_” beside the acyl side chain for PCs, PEs, and TGs refers to acyl chains for which the *sn* position remains to be ascertained. Statistics: difference between WT and KO mice was assessed with a paired Student’s *t* test: *p < 0.05, **p < 0.01 before and $ *p* corr<0.05 after Benjamini-Hochberg correction. Covariance analyses were performed to test the impact of sex, and are represented by colored squares under each lipid.

First and foremost, the hepato-specificity of our KO model was validated by the lack of change in cardiac LRPPRC content, regardless of sex (Fig. 6a). We thereby sought to determine whether the cardiac lipidome was affected considering the fact that hepatic lipid metabolism alterations eventually lead to a remodeling of circulating lipid levels as previously seen with our liver-specific *Lrpprc*-*null* mice^16^. More precisely, this *Lrpprc* KO model also exhibited changes in hepatic and plasma lipidome. In the liver, we previously observed increased TG, GPL, and long-chain acylcarnitines as proxies of impaired hepatic mitochondrial β-oxidation while in plasma DHA-containing PCs and cholesteryl esters were decreased^16^. Figure 6b shows a volcano plot of all 2214 MS features of the data set. Using a subjective corrected *p*-value threshold of 0.1 (corresponding to an uncorrected *p* value of 0.007), 162 features discriminated KO from control mice, of which 46 lipids were subsequently identified by MS/MS (Fig. 6b; Supplementary Fig. 3). Out of 31 glycerophospholipids (GPL) identified, 18 GPLs (Fig. 6c), and 10 ether lipids (Fig. 6d) were decreased (0.30- to 0.88-fold and 0.30- to 0.74-fold, respectively). Changes for some GPLs levels were impacted by sex, with KO females having a greater decrease in phosphatidylcholines (PC) and phosphatidylethanolamines (PE) than their male counterparts (Supplementary Fig. 3). Since GPLs are involved in cardiolipin maturation, targeting, and identifying them was one of our objectives. However, only CL (76:12) was significantly decreased in KO mice (0.59-fold; Fig. 6e), and no sex impact was noted. Finally, 15 glycerolipids were identified (Fig. 6f). Interestingly, triglycerides with up to 58 carbons were all significantly increased in KO females while those with more than 60 carbons were significantly decreased. Although less pronounced, this pattern was also observed in KO males with a significant decrease in longer-chain triglycerides levels (≥60 C), but no significant changes in shorter-chain triglycerides (≤58 C) (Supplementary Data 4). Overall, these results demonstrate significant remodeling of the cardiac lipidome in both sexes with KO females being more affected than their male counterparts.

### Loss of hepatic LRPPRC induces mitochondrial remodeling mainly in females’ hearts

**Fig. 7:**
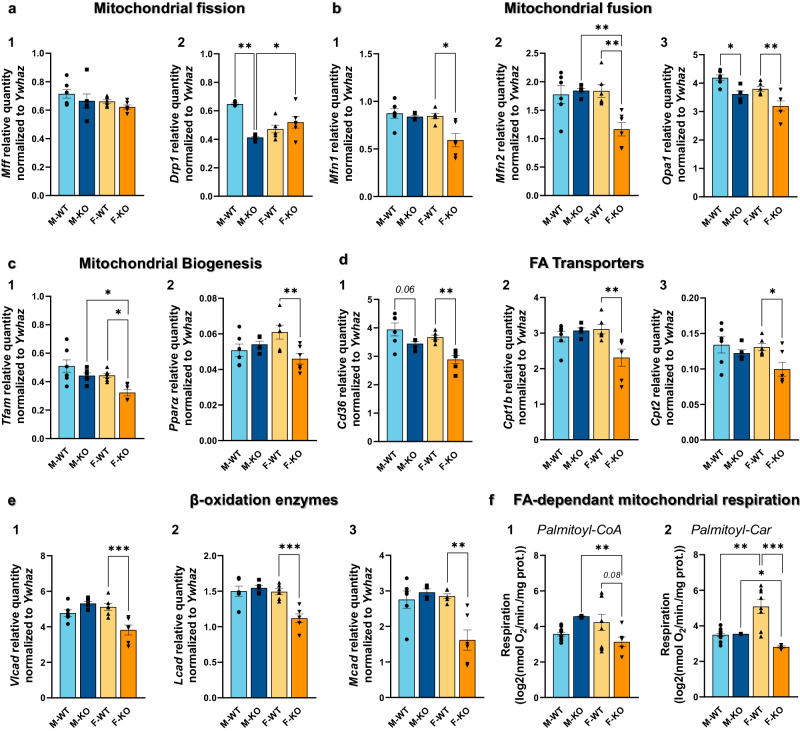
Mitochondrial general profile including biogenesis, fission, fusion, metabolism, respiration in the cardiac tissue of normal and liver-specific LRPPRC deficient mice. At 14 weeks of age, the mRNA levels were quantified for mitochondrial fission markers **a1** *Mff* and **a2** *Drp1*, for mitochondrial fusion markers **b1** *Mfn1*, **b2** *Mfn2*, and **b3** *Opa1*, for mitochondrial biogenesis markers **c1** *Tfam* and **c2** *Pparɑ*, a nuclear receptor functioning as a transcription factor, for FA transporters markers **d1** *Cd36*, **d2** *Cpt1b*, and **d3** *Cpt2*, and for β-oxidation enzymes markers **e1** *Vlcad*, **e2** *Lcad*, and **e3** *Mcad*. At 14 weeks of age, mitochondrial respiration was assessed in the presence of **f1** Palmitoyl-CoA and **f2** Palmitoyl-carnitine. Statistics: two-way ANOVA test followed by Šidák multiple comparison post hoc analysis for normally distributed data in **b2**–**e3**; Kruskal-Wallis ANOVA test followed by Dunn’s uncorrected test for non-normally distributed data in **a1**, **a2**, **b1**, **f1,** **2**. *p < 0.05, **p < 0.01, ***p < 0.001, ****p < 0.0001.

Since an altered mitochondrial metabolism can contribute to a remodeling of different lipid classes and since cardiolipins are a key and essential component for the mitochondrial membrane, the observed changes in the lipidome as well as the significant decrease of cardiolipin 76:12 in KO’s hearts prompted us to look at the mitochondrial life cycle and other important actors in FA utilization and oxidation. We first targeted mitochondrial biogenesis by quantifying mRNA levels of mitochondrial transcription factor A (*Tfam*). As shown in Fig. 7c1, a significant 0.73-fold decrease was only observed in KO females. No changes were found in KO males except for a significant difference with KO females. We then sought to evaluate the impact of hepatic *Lrpprc* deletion on mitochondrial dynamics. The expression of the mitochondrial fission genes *Mff* and *Drp1* was slightly impacted by the hepatic loss of LRPPRC (Fig. 7a1, 2). In fact, no changes were found in the expression of *Mff* whereas *Drp1* was significantly decreased by 0.72-fold in KO males. The expression of mitochondrial fusion genes was, however, significantly impacted. As shown in Fig. 7b1–3, the expression of *Mfn1*, *Mfn2*, and *Opa1* was decreased by 0.70, 0.63, and 0.84-fold, respectively, mostly in KO females except for *Opa1* where KO males also show a significant decrease in mRNA levels. Of note, biogenesis, and fusion markers were presenting a sexual dimorphism in KO mice. These results indicate that hepatic loss of LRPPRC may affect the integrity of cardiac mitochondria predominantly in KO females while less affected in KO males with exceptions for some markers of mitochondrial dynamic.

Given the identification of flaws in mitochondrial dynamics and biogenesis, we proceeded to explore whether there were impairments in fatty acid metabolism in KO mice. We first targeted the nuclear receptor *Pparɑ*, a coordinator of the translational cascade of proteins involved in fatty acid metabolism. As shown in Fig. 7c2, the expression of *Pparɑ* is significantly decreased by 0.75-fold in KO females only. mRNA levels of several β-oxidation transporters and enzymes were also quantified. As such, *Cd36*, *Cpt1b*, and *Cpt2* were decreased by 0.79-, 0.74-, and 0.76-fold, respectively only in the heart of KO females (Fig. 7d1–3) while only *Cd36* tends to be lower in KO males (*p* = 0.06). Consistently with previous results, such as liver transcriptomics, *Vlcad*, *Lcad*, and *Mcad* were significantly decreased only in KO females (0.75-, 0.57-, and 0.75-fold, respectively) (Fig. 7e1–3). Interestingly and consistently with impaired mitochondrial integrity, gene markers of fatty acid transport and oxidation were marked by a strong sexual dimorphism in KO mice. To functionally validate our molecular results, isolated mitochondria from cardiomyocytes underwent fatty acid-stimulated respiration in presence of (i) palmitoyl-CoA, and (ii) palmitoyl-carnitine (Fig. 7f1, 2). Consistent with our results, mitochondrial respiration in female KO was reduced by 0.74-, and 0.58-fold in the presence of palmitoyl-CoA, and palmitoyl-carnitine respectively. As expected, no changes were seen in KO males.

Taken together, these results suggest that *Lrpprc* deficiency has a direct impact on mitochondrial integrity, and fatty acid oxidation capacity predominantly in the hearts of female KO. However, KO males seem to be somewhat protected and/or unaffected by this cardiometabolic impairment. These results are consistent with our lipidomics analysis which demonstrated intracardiac accumulation of certain triglycerides, shorter-chain triglycerides (≤58 C), predominantly in KO females (Fig. 6f).

### Loss of hepatic LRPPRC causes impairment of cardiac diastolic function only in males

**Fig. 8:**
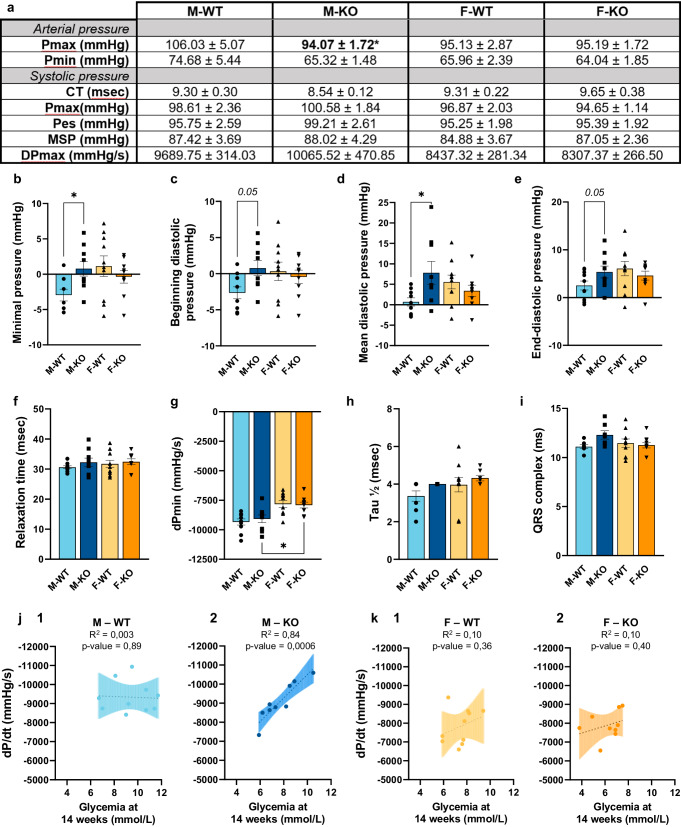
Cardiac parameters assessed by Millar probe in normal and liver-specific LRPPRC deficient mice. **a** Parameters of blood pressure were measured in the right carotid artery (Pmax, maximum pressure; Pmin, minimum pressure). Once entered into the left ventricle, parameters of systolic pressure were acquired (CT, contraction time; Pmax, maximum pressure; Pes, end-systolic pressure; MSP, mean systolic pressure; dPmax, maximum developed pressure). Different parameters of diastolic pressure were also measured and represented by the **b** minimal pressure, **c** beginning diastolic pressure, **d** mean diastolic pressure, **e** end-diastolic pressure, **f** relaxation time, **g** myocardial relaxation index, and **h** Tau ½. **i** Amplitude of the QRS complex obtained through ECG monitoring. Correlations analyses between the myocardiac relaxation index -dp/dt and glycemia carried out with a simple linear regression in (**j1**) WT and (**j2**) KO male mice, (**k1**) WT and (**k2**) KO female mice, *R*^2^ and *p* value are shown in each graph. Statistics: two-way ANOVA test followed by Šidák multiple comparison post hoc analysis for normally distributed data in **b**–**g**; Kruskal–Wallis ANOVA test followed by Dunn’s uncorrected test for non-normally distributed data in (**h**–**i**); simple linear regression and Pearson’s correlation analyses for (**j1**–**k2**). *p < 0.05, **p < 0.01, ***p < 0.001, ****p < 0.0001.

Since the hepatic loss of LRPPRC has an impact on the bioenergetic capacities of KO females, we pursued to see whether this was affecting cardiac function. A Millar catheter was therefore used to acquire the parameters of arterial in the carotid artery and left intraventricular pressure. As shown in Fig. 8a, the maximum arterial pressure (Pmax) was 0.89-fold decreased in KO males compared to their controls. Otherwise, the minimum arterial pressure (Pmin), and every other parameter of systolic function (CT, Pmax, Pes, MSP, dPmax) did not vary in KO mice, and regardless of sex. However, this was not the case for diastolic parameters. In fact, as shown in Fig. 8b, the minimum pressure (0.778 ± 1.047) as well as the beginning-, mean-, and end-diastolic pressures (Fig. 8c–e respectively) were all increased in KO males (0.766 ± 1.035; 6.830 ± 2.224; 6.019 ± 1.178 respectively). No changes were found in KO females. Furthermore, a few other cardiac parameters were analyzed, such as relaxation time, myocardial relaxation index (dPmin) and Tau (Fig. 8f–h respectively). However, these parameters did not exhibit any notable alterations, except for dPmin which displayed a significant disparity between male and female mice, with no dissimilarities between WT and KO. However, ECG results showed no significant differences for the amplitude of the QRS complex, which tended only weakly to an increase of 1.08-fold in KO males (*p* = 0.28; Fig. 8i). Because alterations in diastolic function can be explained by the presence of fibrosis amongst other causes, this hypothesis was addressed. Despite a trend towards increased fibrotic markers in males (*Col1a1*: *p* = 0.1205, and *Col3a1*: *p* = 0.1706; Supplementary Fig. 3a, b) and a significant sex source of variation (*Col1a1*: *p* = 0.0009, and *Col3a1*: *p* = 0.0257), no difference was noted in histological quantification (Supplementary Fig. 3d). No changes were detected in KO females.

Finally, in order to link and analyze the hypoglycemic profile of KO male mice with their above-mentioned diastolic dysfunction, we performed simple linear regression and Pearson correlation analyses between glycemia, and each parameter recorded by Millar catheter. While most of the parameters did not correlate with the hypoglycemia we measured, myocardial relaxation index (dPmin) exhibited a robust and inverse correlation with glycemia, yielding an R-squared value of -0.84 and a p-value of 0.0006 only for KO male mice (Fig. 8j2). Conversely, neither WT males, WT females nor KO females showed a significant correlation (Fig. 8j1, k1, 2 respectively). Altogether, these results point out to a deleterious cardiac phenotype only in KO males through the increase of diastolic pressure parameters and an inverse correlation between myocardial relaxation index and glycemia. KO females did not exhibit any of these observations.

## Discussion

The aim of this study was to characterize the cardiac abnormalities linked to lean-NAFLD and assess the impact of sex using a liver-specific *Lrpprc*-*null* mouse model. *Lrpprc* KO mice were followed from 5 to 14 weeks of age, and evaluated at hepatic, circulating, and cardiac levels. Our results reveal that specific loss of LRPPRC in the liver does not lead to obesity but induces deleterious remodeling of the liver matrix with signs of fibrosis and impaired hepatic structure similar in both sexes recapitulating to some extent the lean-NAFLD phenotype in humans. Importantly, loss of hepatic LRPPRC goes beyond liver damage and results in a pronounced sex-dependent multi-faceted phenotypic remodeling including, only in males, additional defects that include liver macrophage infiltration and increased circulating FGF21 levels. KO mice exhibit disturbances in glucose metabolism with fasting hypoglycemia and decreased insulin levels in both sexes at 9 weeks. Later in age, at 14 weeks, while insulin is still decreased in KO males, no changes are observed in KO females. In addition, changes in insulin sensitivity are predominantly found in KO males. KO mice are displaying cardiac abnormalities, with females showing cardiometabolic impairments, whereas males are manifesting signs of diastolic dysfunction. Finally, diastolic dysfunction in KO males is closely associated with hypoglycemia since there is a strong and negative correlation between fasting glycemia and myocardial relaxation index (-dP/dt). This study gives new insight into the crucial involvement of mitochondria in the pathophysiology of lean-NAFLD and its cardiac impact.

### Loss of hepatic LRPPRC induces liver defects in both KO males and females, although more advanced in KO males

So far, only one study reporting anthropometric parameters has been published showing that, at 5 weeks of age, liver-specific *Lrpprc* KO mice exhibited reduced body weight despite their normal appearance and locomotor activity under normal cage-bound conditions^14^. Consistently, our data showed that in addition to having a reduced weight at 5 weeks, the weight curve was maintained until 14 weeks regardless of sex (Fig. 1c). Interestingly, the hepatic phenotype appeared to have evolved similarly in both males and females from a more steatotic liver at 5 weeks to a more fibrotic and inflamed liver at 14 weeks. Although these are typical histological features of severe mitochondria hepatopathy^24^, increased levels of fibrosis, inflammatory and ER stress markers recapitulates what can be observed in a population with steatohepatitis (NASH), and to some extent, with early stages of cirrhosis^21,25–28^.

However, our data on cortisol concentration shows that KO females had lower circulating levels (Fig. 2f). Based on a study by Ahmed et al.^29^, there are two seemingly protective phases of altered hepatic cortisol metabolism in progressive NAFLD. In steatosis, increased cortisol clearance, thus lowering its local levels, would preserve the hepatic metabolic phenotype and limit lipid accumulation. Conversely, in NASH, increased cortisol regeneration, and thus, higher local cortisol levels would possibly limit hepatic inflammation. Therefore, although it should be kept in mind that these data were generated in an obese population, our results may indicate that the KO female organism attempts to preserve the liver, contributing to the subtle phenotypic contrast between males and females. This could also explain the absence of difference in ALT activity, a classic marker of liver damage, despite KO males displaying a slight trend towards an increase.

Despite similar liver remodeling in both sexes, our transcriptomic data are pinpointing a sexual dimorphism suggesting the possibility of distinct mechanisms in the emergence and/or progression of this liver phenotype (Fig. 4a, b). While KO males showed a decrease in transcripts involved in mitochondrial organization and its phosphorylative capacity, KO females had a more pronounced decrease in transcripts involved in lipid metabolism. Although network modeling for sex differences in NAFLD is still an emerging field, our data are consistent with a recent study pinpointing perturbations of mitochondria and fatty acid biosynthesis gene expression in males and females *Pparγ*-*null* mouse model, respectively, who were fed with chow diet^30^. Kurt et al. showed that oxidative phosphorylation and lipid metabolism were shared between sexes, with males showing prominent lipid-related processes (e.g. phospholipids, lysophospholipids) compared to females^31^. However, since this study was based on the transcriptome of diet-induced NAFLD mice, the development and progression of the disease also depended on adipose tissue dysfunction, knowing that female mice are protected to some extent from dysmetabolism. Our study, on the other hand, describes the transcriptome analysis of a lean-NAFLD mouse model, which contains a sex-dependant signature specific to lean-NAFLD without any sign of confounding systemic effects.

Furthermore, NAFLD/NASH patients often present different extents of dyslipidemia (low level of HDL-C, increased levels of LDL particles and triglycerides), which serve as an important non-invasive marker for NAFLD diagnostics. It has been reported that lean and overweight/obese NAFLD patients share this common lipid profile with both lean and overweight/obese controls^32^. Our results were consistent with this evidence, although only KO male mice exhibited increased LDL-C levels, and decreased HDL-C levels (Fig. 4c). Even though sex was not studied separately, our previous study showed that this *Lrpprc* KO mice model also exhibited plasma lipidome remodeling with a notable decrease in DHA-containing PCs, and cholesteryl esters as well as free DHA^16^. In addition, we previously observed in the liver from *Lrpprc* KO mice increased short-chain TGs glycerolipids, GPLs (including various PCs and PEs) and long-chain acylcarnitines, the latter being consistent with defective hepatic mitochondrial β-oxidation^13^. However, such a lipid profile is considered atherogenic and is also linked to an increased risk of developing cardiometabolic complications^33^. Interestingly, female *Lrpprc* KO mice had a different profile with lower plasma levels of lipoproteins than their controls (Fig. 4d), a pattern that was also observed in their transcriptome (Fig. 4b). Although no analysis of TG influx and cholesterol efflux has yet been performed, these findings suggest that they may have a synthesis defect. Besides, it has been shown that VLDL synthesis and lipid outflow from the liver were impaired in patients with NASH, but not in patients with NAFLD^34^.

Overall, our data suggest the presence of a comparable but distinct liver phenotype across sexes, which recapitulates the damage found in NASH. However, histological data, cortisol levels, transcriptomic data, and total cholesterol in plasma lipoproteins fractions leave room for the possibility of distinct, sex-dependent mechanisms and biomarkers underlying lean-NAFLD progression, which could therefore have an impact on the extra-hepatic manifestations. Similarly, in humans, NASH, a central stage in the progression of NAFLD, is a multi-faceted condition, and pinpointing reliable molecular biomarkers has been challenging. In accordance with this, sexual dimorphism has frequently been observed in the progression of NASH, and it is believed that sex plays a significant role in determining liver response to NASH and its progression in human patients^35^. More precisely, the NAFLD/NASH phenotype tends to be more severe in men than in nonmenopausal women^36^.

### Hepatic LRPPRC deficiency disrupts glucose metabolism predominantly in males

Compared to lean populations, previous studies on lean-NAFLD reported a higher risk of impaired glucose tolerance in mouse models and human cohorts as they either exhibit signs of type 2 diabetes and metabolic syndrome or belong to the phenotype of metabolically obese normal weight [reviewed in refs. ^5,37^]. However, since our liver phenotype shows promising signs of steatohepatitis at 14 weeks, it appears that few studies on lean-NASH have considered its impact on glucose metabolism.

Our data suggests that *Lrpprc* KO mice have a fasting hypoglycemic profile and altered insulin sensitivity, a profile exacerbated in males (Fig. 5a). The fact that males were more affected than females seems to be in agreement with the liver phenotype and histological data where KO males exhibit an exacerbated inflammatory state based on the F4/80 marking (Fig. 2) and FGF21 hepatokine (Fig. 3j), increased ALT activity and no changes in circulating cortisol levels, as detailed above, which would point out to a somewhat more advanced stage of liver disease in KO males. These results have also been observed in a mouse model of methionine-choline deficient (MCD) diet-induced steatohepatitis^38^. However, since little is known about the relationship between lean-NASH and hypoglycemia, we speculate that the underlying mechanisms could be associated with a compensatory system on behalf of the organism.

This hypothesis is based on artificial nutrition procedures where after undernutrition and a prolonged fast, depletion of hepatic glycogen reserves followed by insulin hypersensitivity is often observed^39^. In fact, severe hepatic disturbances would impinge on glycogen storages which would weaken the liver’s capacity to initiate gluconeogenesis during a fasting period. Gluconeogenesis being inhibited by insulin, the latter would become less necessary, and, therefore, less secreted. Our data are in agreement with this postulate since *Lrpprc* KO mice exhibited lower insulin levels at 9 weeks of age regardless of sex (Fig. 5a1). This could also apply to cortisol levels which, although not specific to glycemia control, increases in energy availability by directly inhibiting insulin-producing pancreatic β-cells^40^. To compensate for this insulin decrease and because euglycemia can be primarily maintained by endogenous glucose production in the liver^41^, insulin-sensitive organs and tissues such as skeletal muscles would be forced to adapt by increasing their sensitivity to insulin^42^. Mice would then present a reactive hypoglycemia. However, the response of *Lrpprc* KO mice to a bolus of glucose was not similar to that observed in the severely malnourished. Generally, glucose intake would provoke insulin secretion, thereby leading to a transient correction of hypoglycemia^43,44^. When moderate, this hypoglycemia is often asymptomatic. But, in situations of insulin hypersensitivity, symptomatic hypoglycemia may occur. However, our OGTT data, rather than suggesting distinctive signs of postprandial hypoglycemia, suggest a rapid reuptake (15–30 minutes post-gavage) of glucose by skeletal muscles (Fig. 5d).

It should be noted that during prolonged fasting period—or during energy demand—and once hepatic glycogen storages are depleted, neoglucogenesis uses amino acids, lactate, pyruvate, and glycerol as substrates for de novo glucose synthesis. Known as the Cori cycle, it could explain the struggle to maintain homeostasis between a liver with depleted glycogen reserves, and skeletal muscles that are hypersensitive to insulin and rapidly metabolizing glucose. Thus, an imbalance of metabolic substrates or an alteration in gluconeogenesis signaling pathway can lead to hypoglycemia.

Interestingly, based on the modest decrease in PEPCK content found in KO males (Supplementary Fig. 2b), we can only speculate that this mechanism is a possible driver of hypoglycemia in males. Furthermore, based on the significant decrease in hepatic GS content in KO females (Supplementary Fig. 2a), we hypothesized that they might have a less efficient glycogenolysis. However, given that there was no significant difference in blood glucose levels compared to their controls and that females naturally have a better glucose homeostasis and insulin sensitivity [reviewed in^45^], perhaps they would have a protective mechanism to avoid severe, and potentially fatal, hypoglycemia.

With that being said, any disruption in liver metabolism, structural integrity, or intracellular dynamics may impair the hepatic ability to stabilize normal glucose homeostasis, and hypoglycemia may occur. Other plausible mechanisms would involve (i) hyperglucagonemia, which might induce downregulation of hepatic glucagon receptor or blunt the hepatic counter-regulatory response to hypoglycemic events^46^, and/or (ii) increased oxidative stress, an important determinant of hypoglycemia^47–49^. None of these mechanisms have yet been investigated, and pursuing these avenues of research would help to clarify the link between lean-NAFLD and hypoglycemia.

### Hepatic loss of LRPPRC induces sex-dependent cardiac manifestations

Clear sexual dimorphism exists in cardiometabolic health susceptibility. However, data on the mechanisms underlying the progression of cardiovascular events in the lean-NAFLD population are limited, and even more so when trying to understand the impact of sex. Our study shows that hepatic loss of LRPPRC led to sex-dependent cardiac alterations that affected both mitochondrial/metabolic phenotype, and contractile function. While females presented greater cardiometabolic impairments than males, only the latter showed impaired diastolic function.

Our results suggest that only KO male mice displayed diastolic dysfunction due to increased filling pressure (Fig. 8b–e). This mild diastolic dysfunction could be associated with mitochondrial dysfunction. In fact, however, across all targeted mitochondrial genes in KO males, only the fission marker Drp*1* (Fig. 7a2), and the fusion marker Opa*1* (Fig. 7b3), exhibited decreased levels. This observation implies that while mitochondrial fission and fusion could be affected only slightly in males, the extent of this impact appears to be much less pronounced when compared to females. Interestingly, using cardiac *Drp1* knockout mice, *Song* et al. showed that inhibition of mitochondrial fission, but not fusion, occurred in adult hearts with increased myocardial fibrosis^50^. Moreover, in explanted failing human heart samples and in a rat heart failure model, OPA1 protein level was significantly reduced, even though *Mfn1/2* gene and protein levels remained unchanged^51^. Our results would therefore be consistent with these data when considering KO males, especially since, at the molecular level, we had observed a trend towards an increase in cardiac fibrotic markers (Supplementary Fig. 3). It should be noted that males displayed a tendency towards increased QRS complex amplitude, which could be associated with conductance defects and in cases of advanced pathology and to myocardial scarring resulting from accumulation of fibrotic tissue following cardiac trauma^52^. However, no histopathological signs associated with fibrosis were observed, but this does not exclude the fact that it could have been the case if mice were followed for longer than 14 weeks. On the other hand, hepatic histological data allowed us to carry further our speculation of a more advanced state of hepatic disease in KO males only as they exhibit a more pronounced lack of structure and macrophage infiltration (Fig. 2a, b). In conjunction with mitochondrial dysfunction, remodeling of the circulating and cardiac lipid profile is known to have an impact on the development of cardiac fibrosis^53^. Hence, as described above, remodeling of the circulating lipidome and the increased presence of LDL-C, to the detriment of HDL-C levels (Fig. 4c), would also be involved in the intracardiac increase of deleterious TGs (≤58 C^54^) in KO males (Fig. 6f), and altogether to the development of heart muscle dysfunction in KO males.

Conversely, and despite cardiometabolic impairments, our results in KO female mice showed no evidence of contractile dysfunction. In fact, they exhibited greater cardiac lipidome remodeling than males with, among other species, greater intracardiac accumulation of deleterious TGs (≤58 C) (Fig. 6f). This would be the result of imbalanced lipid metabolism caused by: i) a decrease in reverse cholesterol transport mediated by HDL-C, HDL lipoproteins being decreased, and (ii) a decrease in fatty acid oxidation due to an impairment of β-oxidation (Fig. 7). In addition, they exhibited impaired mitochondrial biogenesis and fusion, as fission was not affected (Fig. 7). Consistently, we observed a decrease in CLs (Fig. 6e)—although sex-independent—which is known to affect fission/fusion processes, and reduce respiratory performance^55^. Since these results are risk factors for cardiovascular damages, this lack of functional impairments at 14 weeks could be explained by the difference in sex steroid hormones. In fact, it has been shown that cardio-specific *Ampkα2* deficiency induces, only in males, left ventricular systolic dysfunction, cardiac fibrosis, and cardiac cardiolipin remodeling^56^. In ovarectomized females, the presence of cardiac fibrosis and mild cardiolipin remodeling was observed, but no cardiac dysfunction was found, suggesting the possibility that males do not benefit from female hormones protection and/or that male hormone may have some deleterious action. In addition, sex hormone deprivation in both male and female rats provided evidence that: (i) sex hormones have a cardioprotective role, especially female ones, and (ii) ovarectomized females showed first signs of cardiac dysfunction at 12 weeks of age^57^. We can therefore speculate that with estrogen deficiency—a hypothesis that we cannot rule out given the impact of estrogen deficiency on mitochondrial dynamics and fatty acid metabolism^58^—*Lrpprc* KO females might develop cardiac dysfunction with or without fibrosis later in life. Furthermore, it is important to mention that alterations of mitochondrial biogenesis, dynamics, and fatty acid metabolism suggest an impairment of mitochondrial integrity in females, a profile that would most likely be exacerbated in the presence of endogenous stress and might as well eventually trigger a functional pathological phenotype.

### Deleterious alterations in liver and heart reveal a sexual dimorphism towards a more severe phenotype for KO males

As mentioned earlier, liver-specific *Lrpprc* KO mice show hepatic structural damage and hepatocellular injury independently of sexes. However, KO males show a more severe and pronounced phenotype when considering the hepatic inflammatory aspect as well as concentrations of circulating hepatokines and cardiac diastolic function. In contrast to KO female mice, our observations in KO male mice can be summarized as follows: (i) a heightened hepatic macrophage infiltration highlighted by increased levels of F4/80 staining (Fig. 2), (ii) increased concentration of FGF21 (Fig. 3) and (iii) a more severe hypoglycemic state that inversely correlates with the impaired myocardial relaxation index (Fig. 8) in the absence of any major cardiac mitochondrial markers remodeling (Fig. 7). Although we are not able to elaborate on the underlying mechanisms driving these sex-dependent outcomes, we can, however, speculate on the above-mentioned evidence corroborating the exacerbated liver disease profile in KO males leading them towards the observed diastolic dysfunction.

Firstly, KO males depicted a more defined and pronounced inflammatory hepatic profile, as highlighted by the F4/80 staining, in favor of an increase in macrophage infiltration and inflammation state that may be associated with the impairment of cardiac diastolic function for which we cannot yet describe nor identify any clear mechanisms. Nonetheless, increased macrophage infiltration is often described to produce hepatic damage with the production of ROS and inflammatory mediators and are thus key actors in NAFLD pathogenesis, which may consequently lead to the progression of a more critical liver disease^59–61^. Consistent with our observations, several evidence support that advanced states of liver disease, with an inflammatory component, are associated with diastolic dysfunction. As such, cirrhosis, the most severe form of liver disease, has been extensively associated with diastolic dysfunction^62–65^. In addition, NASH, has been directly associated with cardiac morphological changes and disturbed diastolic function^66^, and this premise has also been observed in a non-obese population with NAFLD and, more precisely, an association with NAFLD without obesity and decreased E/A (an index of left ventricular relaxation in diastole) has indeed been shown^67^.

Secondly, a significantly higher concentration of circulating FGF21 is observed only in KO males. Interestingly, these elevated circulating levels of FGF21 have been highlighted in NAFLD patients by a meta-analysis where the levels of FGF21 showed one of the best sensitivities to segregate NASH from steatosis patients^68^. Moreover, while studies exploring the direct role of FGF21 in driving cardiac functional changes in NAFLD are limited, increased levels of circulating FGF21 have been observed in several cardiac diseases and have been associated with diastolic dysfunction in both humans and mice^69–71^. FGF21 was also shown to be significantly associated with diastolic dysfunction in heart failure patients with preserved ejection fraction^69^ as well as in heart failure patients with reduced ejection fraction^71,72^. Moreover, the FGF21-FGFR4 signaling pathway is proposed as a novel mechanism driving cardiac hypertrophy in type 2 diabetes^70^. Our observations for KO males are in line with these previous findings and we therefore hypothesize that the advanced damage in both the liver and heart of KO male mice might be interconnected. In other words, the diastolic dysfunction observed in KO males may be correlated with a more advanced state of liver disease.

Thirdly, our H-*Lrpprc*^-/-^ mouse model is characterized by disturbed systemic glucose metabolism and especially a more pronounced hypoglycemic profile as well as insulin sensitivity only in KO males (Fig. 5). Many studies highlighted the numerous negative cardiac alterations ensuing hypoglycemia including myocardial dysfunction and stiffness as well as abnormal cardiac repolarization^73–75^, often described in diabetic patients. In fact, poor glycemic control in diabetic patients correlates with diastolic dysfunction^76–78^ and, in contrast, antidiabetic therapy aiming to improve glycemic control especially using GLP-1 receptor agonists or SGLT2 inhibitors, leads to improvements in diastolic function as well as in left ventricular end-diastolic volume^79,80^. Consistent with the latter, our results show a strong significant inverse correlation between glycemia and the myocardial relaxation index, -dP/dt, illustrating indeed impaired myocardial relaxation shadowed by a lower glycemia (Fig. 8j2). Although this result denotes only a correlation between two factors, it can contribute to explaining the cardiac manifestations in this mouse model as well as its sexual dimorphism.

In contrast, in KO females, we do not observe any macrophage infiltration as per the F4/80 staining (Fig. 2) nor any circulating increase in FGF21 concentration (Fig. 3). However, KO females do, in fact, display well-defined metabolic alterations in the absence of any significant cardiac impairments such as the diastolic dysfunction reported in KO males. In this situation, the timeline for the progression of the pathology would appear to be different/delayed between both sexes. We, therefore, cannot exclude the possibility that KO female mice will eventually develop cardiac functional manifestation as a consequence of the observed metabolic disruptions if studied for a longer period of time than 14 weeks, such as in a longitudinal study. Despite our findings not being able to comprehensively grasp the situation and/or its mechanisms, they do, on the other hand, reveal an important and distinct pattern in the development and phenotype of the pathology between males and females. These distinct observations suggest that the progression of the liver disease as well as the important hypoglycemic state, are well connected to the occurrence of diastolic dysfunction more than to the myocardial metabolic dysfunction per se.

## Conclusion

Using a mouse model that has been previously characterized by its mitochondrial hepatopathy in the absence of obesity and recapitulating, to some extent, the circulating lipidomic signature described in NAFLD, we uncovered a sex-dependent multi-faceted extra-hepatic phenotypic remodeling. Males, slightly more than females, exhibited fasting hypoglycemia and increased insulin sensitivity, reflecting altered glucose metabolism potentially caused by dyshomeostasis in the liver-peripheral organ axis. KO males also exhibited mild diastolic dysfunction supported by the presence of mild cardiac mitochondrial dysfunction, remodeling of the circulating lipoprotein profile, and to some extent cardiac lipidome. Overall, these results point to a profile that is typically atherogenic and increases the risk of cardiovascular complications. Interestingly enough, females did not manifest cardiac dysfunction, yet still displayed cardiometabolic impairments. This evidence was supported by impaired mitochondrial integrity and β-oxidation, alongside a decreased circulating lipoprotein profile which all together led to intracardiac accumulation of deleterious triglycerides. However, as a potential explanation aiming to account for this disparity between both sexes, we hypothesize that sex hormones merely postponed the emergence of functional complications. Collectively, these results indicate that metabolic anomalies in the liver can lead to significant sex-dependent abnormalities that affect both the mitochondrial/metabolic phenotype and contractile heart function, independent of obesity. This experimental model may prove useful to better grasp the mechanisms underlying the sex-related variability in the progression of lean-NAFLD in humans.

## Limitations

Our findings demonstrate differences in the development of the pathology between female and male mice, including differences in the rate of pathology progression. Yet, we are not at this stage able to comprehensively explain the mechanisms underlying the discrepancies in terms of cardiac phenotype between male and female mice. It is possible that this pathology progresses more rapidly in male mice compared to female mice. Consequently, female mice may experience prolonged periods of metabolic dysfunction before eventually developing cardiac diastolic dysfunction, whereas male mice may experience diastolic dysfunction at an earlier stage. We, however, speculate that estrogen would serve as a counterbalance and that there would be a fine line between the basal state described in this study and a state induced by endogenous stress. Further investigations are needed to assess the impact of estrogen through methods such as ovariectomy and/or 17 β-estradiol supplementation. Additionally, it is essential to evaluate the impact of endogenous stress (e.g., transverse aortic constriction (TAC) procedure, high-fat diet). Furthermore, the mice study model used in this study relies solely on a mitochondrial dysfunction to replicate NAFLD with a lean phenotype. Since the role of mitochondrial dysfunction in NAFLD is not unequivocally established and isn’t the only contributing factor to the disease’s development and progression, this model indeed requires further validation and analysis for its representativeness in modeling human NAFLD. Nevertheless, we firmly believe that our model offers a unique opportunity to study the multi-organ detrimental effects occurring outside the liver in an organism where hepatic dysfunction is isolated and forms the foundation for other damages, such as what is observed in NAFLD, especially as there is no such model so far. Moreover, it is well known that measuring total food intake per day does not eliminate the variability induced by food intake on metabolic parameters and gene expression. We are aware that some measurements were made while mice were in a stimulated (fed) state (e.g. lipoproteins profile and hepatic and cardiac genes), and therefore not in a homeostasis steady state. However, these experiments were unplanned, exploratory, and performed with the equipment available to us at the time of this study. All our samples were collected by trying to adjust as much as possible the time variable between each group. Therefore, and considering the results presented in this study, these measurements should be repeated in a steady (fasted) state. Since metabolism is not necessarily associated with it and vice versa, it would be interesting to look at growth hormones and to what extent LRPPRC plays a direct role in delayed growth. Finally, given the distinct trajectories of disease progression in males and females presented by our model, a longitudinal study would be strongly required in the future to comprehensively encompass the progression and pace of the pathology as well as the timing of metabolic/molecular and functional defects in both sexes.

## Methods

### Animal model

The following study protocol was approved by the animal research and ethics committee of the Montreal Heart Institute. All experiments were conducted in accordance with the Canadian guidelines for care and use of experimental animals. Liver-specific *Lrpprc-null* mice (H-Lrpprc^−/−^, KO) were produced as previously described in our recent study^14^. Mice were maintained in a specific pathogen-free facility on a regular 12 h light/12 h dark cycle. All mice had access to a standard chow diet and water *ad libitum*. Animals (50% males, 50% females) were weighed, and monitored for their caloric intake once a week. At 14 weeks of age, mice used for experiments were euthanized under isoflurane by exsanguination through the thoracic aorta preceding heart removal. Blood was collected from non-fasted mice in EDTA-coated syringes. Plasma was recovered followed by centrifugation (2000 × *g*, 15 min, 4 °C), and stored at −80 °C in anticipation of further experimentations. The heart, liver, perigonadal white adipose tissue (WAT), interscapular brown adipose tissue (BAT), gastrocnemius, soleus, and tibia were collected, rinsed in cold 0.9% saline, weighted, flash frozen in liquid nitrogen, and stored at −80 °C until subsequent experiments as well.

### Histology

#### Hematoxylin-phloxine-saffron

For general histopathological and morphological assessment, livers were rapidly excised and fixed in 10% formalin PBS-buffered solution for 5 days. Tissues were dehydrated by incubation in a series of solutions with increasing ethanol content (70% to 95% to 100%) followed by xylene and embedded in paraffin^81^. Samples were cut into 6-µm sections and processed with Hematoxylin-Phloxine-Saffron (HPS) staining, commonly used for histological evaluation of liver injury and/or damage. HPS-stained slides were examined by bright-field microscopy (BX45, Olympus, Richmond Hill, ON, Canada), and images were acquired under both ×10 and ×20 magnification. For each slide, 3 different lobules were taken centering on the central vein. The aim was to identify, by qualitative analysis, the possible presence of histopathological signs. The most representative image of each group was chosen for publication.

#### Oil Red’O staining

To quantify steatosis, serial cryosections of 10-µm thickness were taken from livers. On frozen sections, slides were fixed in 10% formalin, incubated in propylene glycol, and processed with Oil Red’O 0.7% (ORO) staining for 2 days. After washing with a series of solutions with a decreased propylene glycol content (100% to 85%), liver cryosections were briefly processed in Mayer’s hematoxylin solution (Leica Biosystems Inc., Buffalo Grove, IL). Following washing, slides were mounted in a glycerin jelly medium. ORO slides were examined by bright-field microscopy (BX45, Olympus, Richmond Hill, ON, Canada), and images were acquired under ×20 magnification. For each slide, 3 different lobules were taken centering on the central vein. Lipid droplet number and morphology in individual hepatocytes were quantified using *ImageJ* (NIH). The most representative image of each group was chosen for publication.

#### Masson’s trichrome staining

To quantify heart fibrosis, hearts were paraffin-embedded, and transverse sections (6-µm) of the ventricles were prepared and stained with Masson’s trichrome solution^82^. A blind analysis of one section per heart was performed by bright-field microscopy (BX45, Olympus, Richmond Hill, ON, Canada), and full heart images were acquired under ×10 magnification. Collagen content was quantified by color segmentation using ImageJ (NIH) and expressed as a percentage of the surface area.

#### F4/80 staining

To better grasp liver inflammation and quantify the area occupied by macrophage infiltration, we targeted the F4/80 molecule which is established as a unique marker of murine macrophages. Liver tissue sections were obtained by the same processes as the HPS staining, through formalin-PBS fixation and paraffin embedment. Immunohistochemistry (IHC) was accomplished using a recombinant rabbit monoclonal F4/80 antibody and anti-rabbit IgG H&L (HRP) antibody used as primary and secondary antibody, respectively. Hematoxylin was used as counterstain for F4/80 slides. The slides were then examined by bright-field microscopy (BX45, Olympus, Richmond Hill, ON, Canada), and images were acquired under both ×10 and ×20 magnification. F4/80 antibody area coverage and general morphology in individual slides were quantified using Image-Pro Premier (9.3). The most representative image of each group was chosen for publication.

### Glucose metabolism

#### Glycemia and insulinemia monitoring

Blood glucose was monitored using a glucometer at 5, 7, 9, 11, and 13 weeks of age at 2 pm following a 5 h food withdrawal. Blood samples (100 µL) were collected at 9, and 14 weeks of age to quantify insulinemia based on the manufacturer’s protocol (Rat/Mouse Insulin ELISA Kit; cat. EZRMI-13K; Millipore Sigma).

#### Insulin tolerance test (ITT)

ITT was performed at 11 weeks of age. Biosynthetic human insulin (Humulin R U-100; Eli-Lilly, Indianapolis, IN; 0.6 U/kg body weight) was injected intraperitoneally in conscious mice at 2 pm after 5 h food withdrawal. Blood glucose was measured using a glucometer at 0, 15, 30, 45, 60, 90, and 120 minutes after insulin administration. The AUC was calculated.

#### Oral glucose tolerance test (OGTT)

OGTT was performed at 12 weeks of age. Glucose (dextrose solution, 2 g/kg body weight; Hospira Inc., Lake Forest, IL) was administered orally by gavage in conscious mice at 9:30am after a 16 h fast. Blood glucose was measured using a glucometer at 0, 15, 30, 45, 60, 90, and 120 minutes after glucose administration. Blood samples were taken from the lateral saphenous vein prior to the gavage (0 minute), and at 30 minutes. Subsequent samples were processed to quantify insulinemia based on the manufacturer’s protocol (Rat/Mouse Insulin ELISA Kit, cat. EZRMI-13K, Millipore Sigma). AUC was calculated.

### Total cholesterol in plasma lipoprotein profile

Plasma of 14-week-old mice were used to measure total cholesterol in plasma lipoprotein fractions. Lipoproteins were separated from plasma by size exclusion chromatography fast protein LC (FPLC) using a Superose 6 column on an FPLC system with a Model 500 pump from Waters (Milford, MA). In short, and as described in a previous study^83^, a 100 μL aliquot of mouse plasma pooled equally from four different mice per group was injected into a 1.0-ml sample loop and separated with a buffer (0.15 M NaCl, 0.01 M Na_2_HPO_4_, and 0.1 mM EDTA) at a flow rate of 0.5 mL/min. Sixty fractions of 300 µL were collected, each containing its correspondent lipoproteins. Batch analysis was performed to measure circulating total cholesterol (Fujifilm Medical Systems U.S.A., Inc. cat. 999-02601) in plasma lipoprotein fractions according to the manufacturer’s protocol.

### Cardiac function assessment using Millar catherization

Fed mice were anesthetized with 2% isoflurane in 100% O_2_ at a flow rate of 2 L/min at 14 weeks of age. Body temperature was monitored and maintained at 37 °C using a heating pad. Cardiac hemodynamics parameters were measured using a microtip pressure transducer catheter (1.4-F; Millar Instruments) inserted into the left ventricle through the carotid artery as previously described^84^. Data were collected and analyzed using IOX software (v.2.10.8.25; emka TECHNOLOGIES, Falls Church, VA). Blood pressure, intraventricular pressures (systole, diastole), and heart rate (HR) measurements were taken when a steady and average value was reached. Subsequent mice were euthanized, and organs were harvested as described in the *animal model* section.

### Cardiac untargeted lipidomic using LC-QTOF

Untargeted lipidomic analyses using LC-QTOF were performed using a previously validated workflow^16,85^. Briefly, lipids were extracted from crushed heart tissues (~50 mg/sample) to which were added six internal standards (LPC 13:0, PC14:0/14:0, PC19:0/19:0, PS12:0/12:0, PE17:0/17:0, PG15:0/15:0; Avanti Polar Lipids Inc, Alabaster, USA). Following lipids extraction, samples (0.7 µL for positive mode, and 2 µL for negative mode) were injected into a 1290 Infinity HPLC coupled to a 6530 accurate mass QTOF MS system equipped with a dual electrospray ionization (ESI) source. Elution of lipids was assessed on a Zorbax Eclipse plus column (C18, 2.1 × 100 mm, 1.8 µm, Agilent Technologies Inc.) maintained at 40 °C using an 83 min chromatographic gradient of solvent A (0.2% formic acid and 10 mM ammonium formate in water), and B (0.2% formic acid and 5 mM ammonium formate in methanol/acetonitrile/methyl tert-butyl ether [MTBE], 55:35:10 [v/v/v]). Samples were analyzed in positive scan mode. MS data were processed using the Mass Hunter Qualitative Analysis software package (version B.06.00, Agilent Technologies Inc.), and a bioinformatics pipeline that we have developed as previously described^85^. Lipid final annotation was achieved using MS/MS analysis on all discriminant MS features. Based on their structures, lipids were annotated by (i) searching the mass-to-charge ratio of selected features in METLIN database and (ii) identifying the polar head and acyl chains based on their signature fragment ion on the MS/MS spectra. Covariance analyses were performed to test the impact of sex.

### RNA-sequencing analyses

RNA-sequencing data were generated at StemCore Laboratories (Ottawa Hospital Research Institute, ON) from livers.

#### Library quantification and construction

Quantification of input Total RNA samples (300–1000 ng of total RNA) was performed with the Qubit HS RNA assay (Thermo Fisher Scientific, Waltham, MA), and the fragment size was evaluated with the Fragment Analyzer HS NGS assay (Agilent, Santa Clara, CA). An RNA Quality Number (RQN) of 8.0 or higher was considered satisfactory quality for library construction. A total of 40 RNA samples were submitted for RNA-seq, and library construction was performed with the Truseq stranded mRNA (Illumina, San Diego, CA). Libraries were prepared with unique barcodes compatible with the Illumina NextSeq 500 platform and were quantified with the Qubit HS RNA assay. Library fragment size was evaluated preceding normalization to a unique concentration. Samples were subsequently pooled in equal amounts.

#### Next generation sequencing

The library pools were diluted to achieve maximal cluster density and ran on 5× High Output Flow Cells on the NextSeq 500. PhiX was spiked in as a control. The samples underwent 1 × 75 cycles of single-end sequencing. This approach yielded up to 50 million reads per sample prior to filtering.

*Analyses*—from the list of non-filtered transcripts reported, those that were below the detection threshold for all conditions were excluded. Transcripts that were below the detection threshold in at least 50% of the mice in the control condition were also excluded. Multiple unpaired *t* tests followed by a false discovery rate (FDR) multiple comparison post hoc analysis (Two-stage step-up (Benjamini, Krieger, and Yekutieli)) were performed for each comparison. Transcripts were considered a discovery when *q*-value < 0.05 (desired FDR (Q) < 5%). Transcripts were classified as up or downregulated based on their log2 fold-change (FC). For each comparison, the Cytoscape (version 3.9.1) ClueGO v2.5.9 plugin^86^ was used to cluster transcripts significantly enriched. A functional group network was therefore generated following enrichment analyses of GO terms (Molecular Function, Cellular Component, and Biological Process) and pathways (KEGG database). The generated clusters expose the relationships between the terms based on the similarity of their associated genes. To obtain these detailed pathways, the GO tree interval was set at 7–15 with a minimum of 2 transcripts from the uploaded list that were (i) associated with a GO term, and (ii) represented at least 20% of the total number of transcripts of the GO term. The degree of connectivity between terms was automatically calculated using the kappa score set at 0.5 in this study. The size of the nodes reflects the statistical significance of the terms, and the ClueGO plugin automatically generates cluster labels based on the most significant term of a group. Therefore, the node color shows the proportion of transcripts from each cluster that are associated with the term. A classical Fisher’s exact test followed by a Bonferroni step-down multiple comparison post hoc analysis was performed by default to show the significance of terms and groups. To ensure that the results are more readable, only pathways with *p* value ≤ 0.05 are shown. Images from Fig. 4a, b were newly created from our RNA-sequencing data.

### Respirometry on isolated cardiac mitochondria

As previously described by Cuillerier et al.^14^, cardiac mitochondria were isolated, and respiration was measured using Clark-type electrodes at 23 °C while stirring continuously. Mitochondria (0.5 mg prot./mL) were suspended in respiration buffer (in mM: 10 KCl, 5 K_2_HPO_4_, 10 MOPS, 9 Pi, 2,5 MgCl_2_, 1 mg/mL BSA; pH 7,4). Following baseline recording, oxidation of long-chain fatty acids was measured, followed by sequential supplementation with (i) palmitoyl-carnitine (20 µM) and (ii) palmitoyl-CoA (5 µM) and L-carnitine (5 mM). Both supplementations were in the presence of malate (2.5 mM) and ADP (1 mM).

### Molecular analyses

#### Quantitative RT-PCR

Gene transcript levels from frozen liver and heart tissues were assessed as previously described for hearts^16,84^ using a RNeasy kit (QIAGEN), and reverse transcribed with the High-Capacity cDNA RT kit (Thermo Fisher Scientific) as recommended by the manufacturer. Quantification was achieved by MxPro software (Agilent Technologies Inc.) and reported using the ΔΔCT method. Gene transcript levels were normalized to *Ywhaz* and *Tbp* in hearts and livers, respectively. The list of primers used is reported in Supplementary Table 1.

#### Immunoblotting

Protein levels from frozen liver tissues were assessed following the loading and running of 20 µg of protein per sample on 12.5% polyacrylamide gels. Samples were transferred to nitrocellulose membrane and used for the detection of LRPPRC (1:1000; LRP130 Rabbit Ab, cat. PA5-22034, Thermo Scientific), MTCO1 (1:10 000; Anti-MTCO1 antibody [1D6E1A8], cat. ab14705, Abcam), Glycogen Synthase (1:1000; Glycogen Synthase (15B1) Rabbit mAb, cat.3886 S, Cell Signaling), and PEPCK (1:1000; PEPCK (H-300) Rabbit polyclonal IgG, cat. sc-32879, Santa Cruz Biotechnology). β-actin (1:50 000; β-actin HRP (C4), cat. sc-47778, Santa Cruz Biotechnologies) was used for normalization. The Precision Plus Protein Dual Color Standards (Bio-Rad, #1610374) were used as a size ladder.

#### ELISA and enzyme activity assays

Levels of plasma cortisol (Cortisol (human/mouse/rat); cat. K7430-100; Biovision), FGF21 (Mouse FGF21 ELISA Kit (ab212160)), RBP4 (RBP4 ELISA Kit (ab195459)) and fetuin A (Mouse Fetuin A ELISA Kit (ab205074)) ELISA Kits and ALT (ALT Activity Assay, cat. MAK052, MilliporeSigma), and AST (AST Activity Assay Kit, cat. MAK055, MilliporeSigma) activity assays were measured spectrophotometrically with the Agilent BioTek Synergy HTX Multi-Mode Microplate Reader from Fisher Scientific. Concentrations used for standard coupled enzyme assays were according to the manufacturer for cortisol, FGF21, RBP4, and fetuin A and adapted for ALT (1:5), and AST (1:10)^87^.

### Statistics and reproducibility

Unless indicated otherwise, data are displayed as mean ± standard error of the mean (SEM). All data and samples (*n* = 6–10/group) were tested for normal distribution. The normality of a data distribution was assessed by the Anderson-Darling normality test. Following this analysis, for normally distributed samples, a two-way analysis of variance (ANOVA) followed by Šidák multiple comparison post hoc analysis was used to determine significant differences in the effects of the genotype and sex variables on the dependent variable. For non-normal samples and for smaller sample sizes, non-parametric analysis was performed using the Kruskal–Wallis test as the one-way ANOVA followed by the uncorrected Dunn’s test. Correlation results were obtained through simple linear regression and Pearson’s correlation analyses. Statistically significant differences were considered for a *p* value < 0.05. All analyses were performed using GraphPad Prism 9.4.0.

### Reporting summary

Further information on research design is available in the Nature Portfolio Reporting Summary linked to this article.

### Supplementary information

Supplementary information

Description of Additional Supplementary Files

Supplementary Data 1

Supplementary Data 2

Supplementary Data 3

Supplementary Data 4

Supplementary Data 5

Reporting Summary

## Data Availability

All data supporting the findings of this study are available within the paper and its Supplementary Information. Uncropped and unedited blot photos with their aligned size markers can be found in the Supplementary Information PDF file, supplementary Figs. 4–6. RNA-sequencing data can be found in the Supplementary Data 1 Excel file as well as deposited on NIH’s sequence read archive (SRA), submission code: SUB14265919, and BioProject ID: PRJNA1079766.
