# Supplementary material for: A genetic mouse model of lean-NAFLD unveils sexual dimorphism in the liver-heart axis
Source: Commun Biol. 2024 Mar 22;7:356. doi: 10.1038/s42003-024-06035-6 (PMC10959946; doi:10.1038/s42003-024-06035-6)
Supplement: Supplementary file 1 — Supplementary information [file 42003_2024_6035_MOESM1_ESM.pdf]

Supplementary Table 1: List of murine oligonucleotides used for RT-qPCR.

Gene	Primer (sense)	Primer (anti-sense)
Hepatic and cardiac remodeling		
Col1a1	CTGCTGGTCCTGCTGGTC	CCTTGTTCGCCTGTCTCAC
Col3a1	CCACAAGGATTACAAGGCATAC	AGGAGCACCGACTTCACC
Tgfb1	GAGAAGAACTGCTGTGTGC	GGTTGTGTTGGTTGTAGAGG
Mmp2	ACACTGGGACCTGTCACTCC	GGGTATCCTCGCTCCAGAGT
Ccl2	CCCAATGAGTAGGCTGGAGA	TCTGGACCCATTCCTTCTTG
Ddit3 (Chop)	GGAAACGAAGAGGAAGAATC	ATAGAACTCTGACTGGAA
Hspa5 (Grp78)	TGCAGCAGGACATCAAGTTC	TTTCTTCTGGGGCAAATGTC
Mitochondrial biogenesis and dynamics genes		
Tfam	GCTGATGGGTATGGAGAAG	GAGCCGAATCATCCTTTGC
Mff	TCCCTTTAAACCTCTGGCACT	CGTTTCTGACCAACTGTCCA
Dnm1l (Drp1)	TGATGGGAAGGGTTATTCCA	TTGGCCAGAGATGGGTACTT
Mfn1	GACCGAAGGGTCAGATGAAA	AGAGGGCACATTTTGCTTTG
Mfn2	TCCCTCGACAGTGTTTCTCC	CAGGTGTCAGAGGGAGAGGA
Opa1	GGAAGAATCGGACCCAAGAG	GGTTCTTCCGGACTGTGGTA
Fatty acid metabolism genes		
Pparα	CAACATGAACAAGGTCAAGGC	GGCAGCAGTGGAAGAATCG
Cd36	AGAGGTCCTTACACATACAG	CTACAGCCAGATTCAGAAC
Cpt1b	ACCAGTCTTAGCCTCTACG	TGTAGCCCAGGTGAAAGG
Cpt2	TGCTCCGAGGCGTTTGTCAGGG	GAGACATTGCAGCCTATCCAGT
Mcad	TTGACGGAACAGCAGAAAG	CCATACGCCAACTCTTCG
Lcad	ATGCCCTATATTGCGAATTACG	CCTTGCTTCCATTGAGAATCC
Vlcad	GGCTCTCCAAGGCTGTATG	ACCACTGCGACTTAACTCTG
Inflammatory genes		
Tnfα	CTATGTCTCAGCCTCTTCTC	CATTTGGGAACTTCTCATCC
Il1β	GGATGATGATGATAACCTGC	CATGGAGAATATCACTTGTTGG
Il6	AAGAAATGATGGATGCTACC	GAGTTTCTGTATCTCTCTGAAG
Housekeeping genes		
Tbp	AAAGACCATTGCACTTCGTG	GCTCCTGTGCACACCATTTT
Ywhaz	AGACGGAAGGTGCTGAGAAA	GAAGCATTGGGGATCAAGAA

Abbreviations: *Col1a1* Collagen Type I Alpha 1 Chain; *Col3a1,* Collagen Type III Alpha 1 Chain; *Tgfb1,* Transforming Growth Factor Beta 1; *Mmp2,* Matrix Metallopeptidase 2; *Ccl2,* C-C Motif Chemokine Ligand 2; *Ddit3,* DNA Damage Inducible Transcript 3; *Hspa5*; Heat Shock Protein Family A (Hsp70) Member 5; *Tfam*, mitochondrial transcription factor 1; *Mff*, Mitochondrial Fission Factor; *Dnm1l (Drp1)*, Dynamin-related protein 1; *Mfn1*, Mitofusin 1; *Mfn2*, Mitofusin 2; *Opa1*, OPA1 Mitochondrial Dynamin Like GTPase; *Pparα*, peroxisome proliferator-activated receptor alpha; *Cd36,* CD36 Molecule, *Cpt*, Carnitine Palmitoyltransferase; *Acadm*, medium- chain acyl-CoA dehydrogenase; *Lcad*, long-chain acyl-CoA dehydrogenase; *Vlcad*, very long-chain acyl-CoA dehydrogenase; *Tnfα*, Tumor necrosis factor α; *Il1β*, Interleukin-1 beta; *Il6*, Interleukin 6; *Tbp*, TATA-Box Binding Protein; *Ywhaz,* Tyrosine 3-Monooxygenase/Tryptophan 5-Monooxygenase Activation Protein Zeta.

Supplementary Table 2 – Organ mass normalized to body weight (BW) in WT and *Lrpprc* KO mice.

Mass / BW	M-WT	M-KO	F-WT	F-KO
**Liver**	**50.14***	46.10	**44.41***	43.87
**Heart**	**4.009*†**	3.983	**4.311*†**	4.420
**WAT**	**10.33*†**	8.718	**4.958*†**	5.339
**BAT**	4.928	5.006	4.873	4.458
**Soleus**	0.407	0.381	0.448	0.445
**Gastrocnemius**	6.216	6.233	6.537	6.268

Statistical analyses were performed using an ordinary two-way ANOVA, and data were corrected for multiple comparisons using, as recommended, Šidák multiple comparison correction (with a single pooled variance). Significance was reached for: *M-WT *vs* F-WT, †M-KO *vs* F-KO. Related to figure 1G.

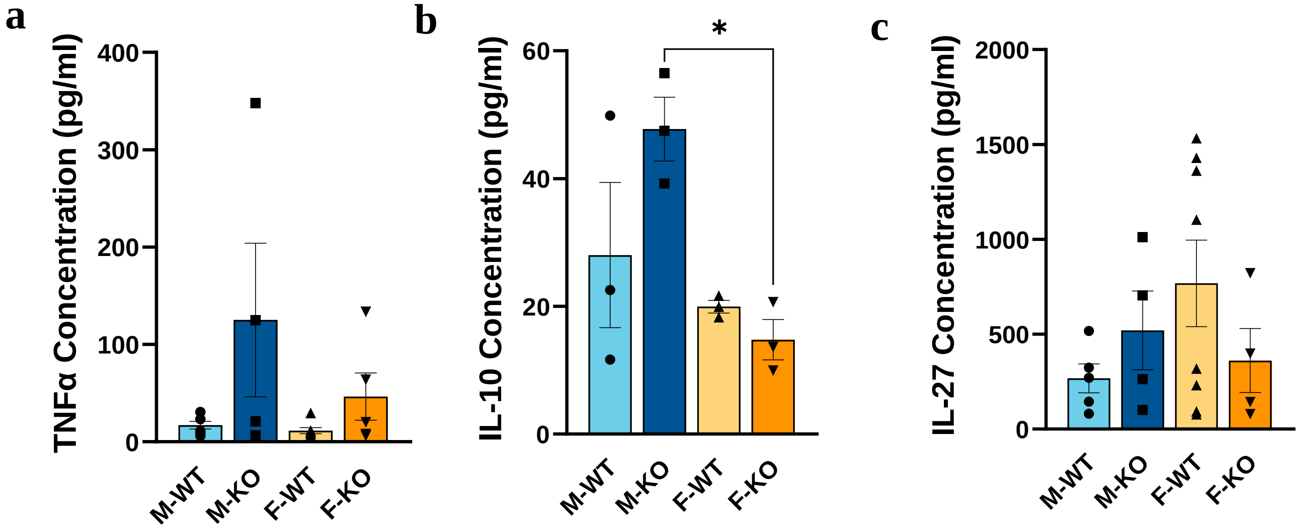

Supplementary Figure 1 – Multiplex readings of circulating cytokines. From plasma collected at 14 weeks of age, several cytokines were tested for their respective concentration. **(a)** Tumor necrosis factor α (TNFα), a cytokine able to promote insulin resistance. **(b)** Interleukin 10 (IL-10), an anti-inflammatory cytokine. **(c)** Interleukin 27 (IL-27), a cytokine regulating the activity of B and T lymphocytes. For normally distributed data, differences between WT and KO mice were assessed with two-way ANOVA tests followed by Šidák multiple comparison post hoc analysis **(b)(c)**. For non-normally distributed data, differences between groups were assessed with the Kruskal-Wallis test (one-way ANOVA) followed by the uncorrected Dunn's test **(a)**. For each statistical analysis: * $p<0.05$, ** $p<0.002$, *** $p<0.001$, **** $p<0.0001$.

Supplementary Table 3 – Transcripts significantly up- and down-regulated based of Log2(fold-change) in females KO *vs.* males KO. Related to figure 3.

mgi_symbol	ensembl_gene_id	q values†	Log2(FC KOF/KOM)
Cyp2c23	ENSMUSG00000025197	0,049	0,911
Gm49431	ENSMUSG00000116130	0,048	1,223
Iigp1	ENSMUSG00000054072	0,046	1,034
Arhgap21	ENSMUSG00000036591	0,038	1,343
Smarca2	ENSMUSG00000024921	0,038	0,605
Srd5a1	ENSMUSG00000021594	0,018	1,662
C6	ENSMUSG00000022181	0,017	2,358
Lifr	ENSMUSG00000054263	0,007	1,226

†Multiple unpaired t-tests were performed using an FDR < 0.05 with the two-stage step-up method of Benjamini, Krieger and Yekutieli.

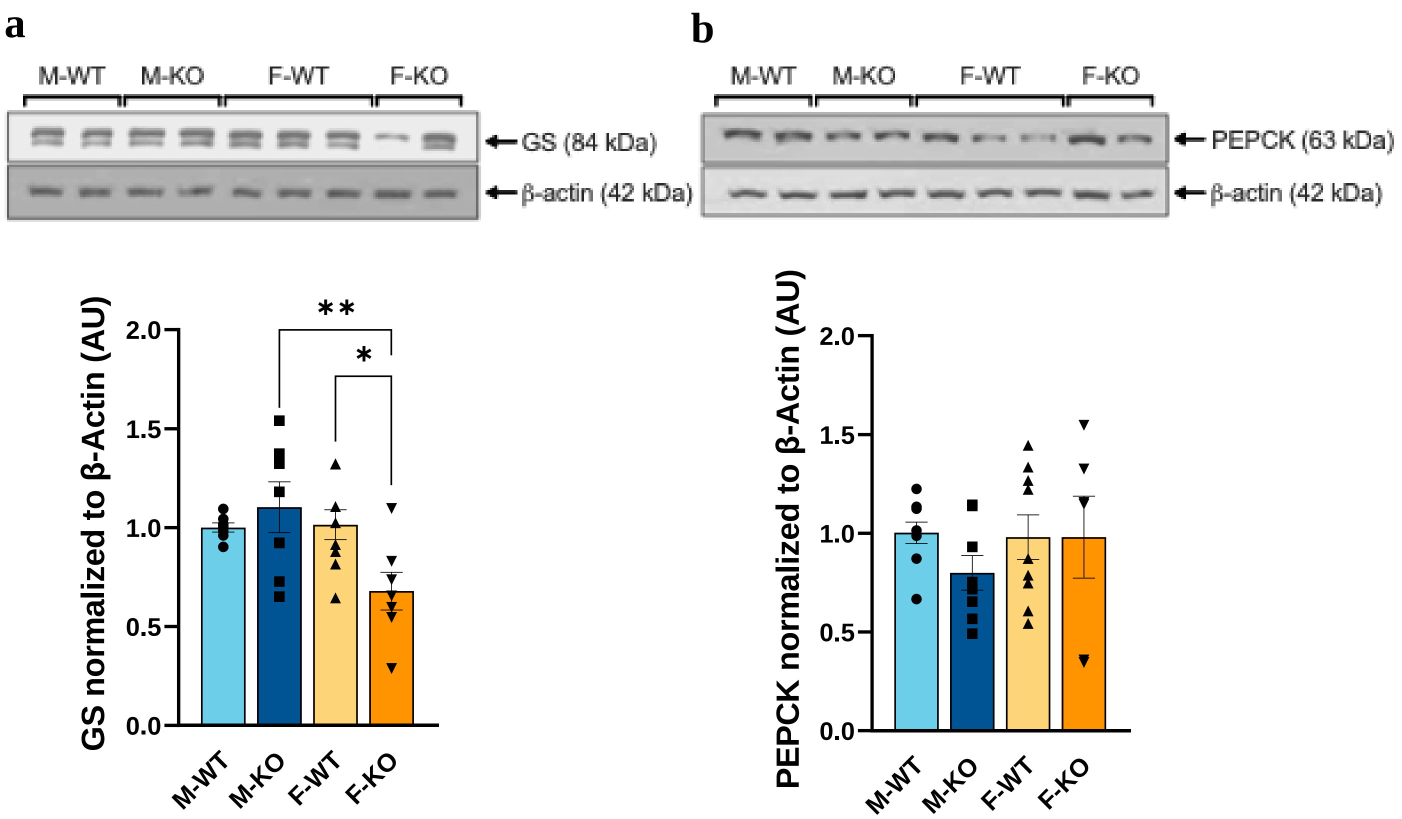

Supplementary Figure 2 – Immunoblot analysis of Glycogen Synthase and PEPCK content in the liver of WT and *Lrpprc* KO mice. Representative images and quantification (box plots; n=10) of **(a)** Glycogen Synthase (GS), and **(b)** PEPCK. β – actin was used as loading control. You can find the uncropped/unedited immunoblots for cardiac each protein content and their aligned size markers in Supplementary Figure 13. Difference between WT and KO mice was assessed with a two-way ANOVA followed by Šidák multiple comparison post hoc analysis: * p<0.05, ** p<0.002, *** p<0.001, **** p<0.0001.

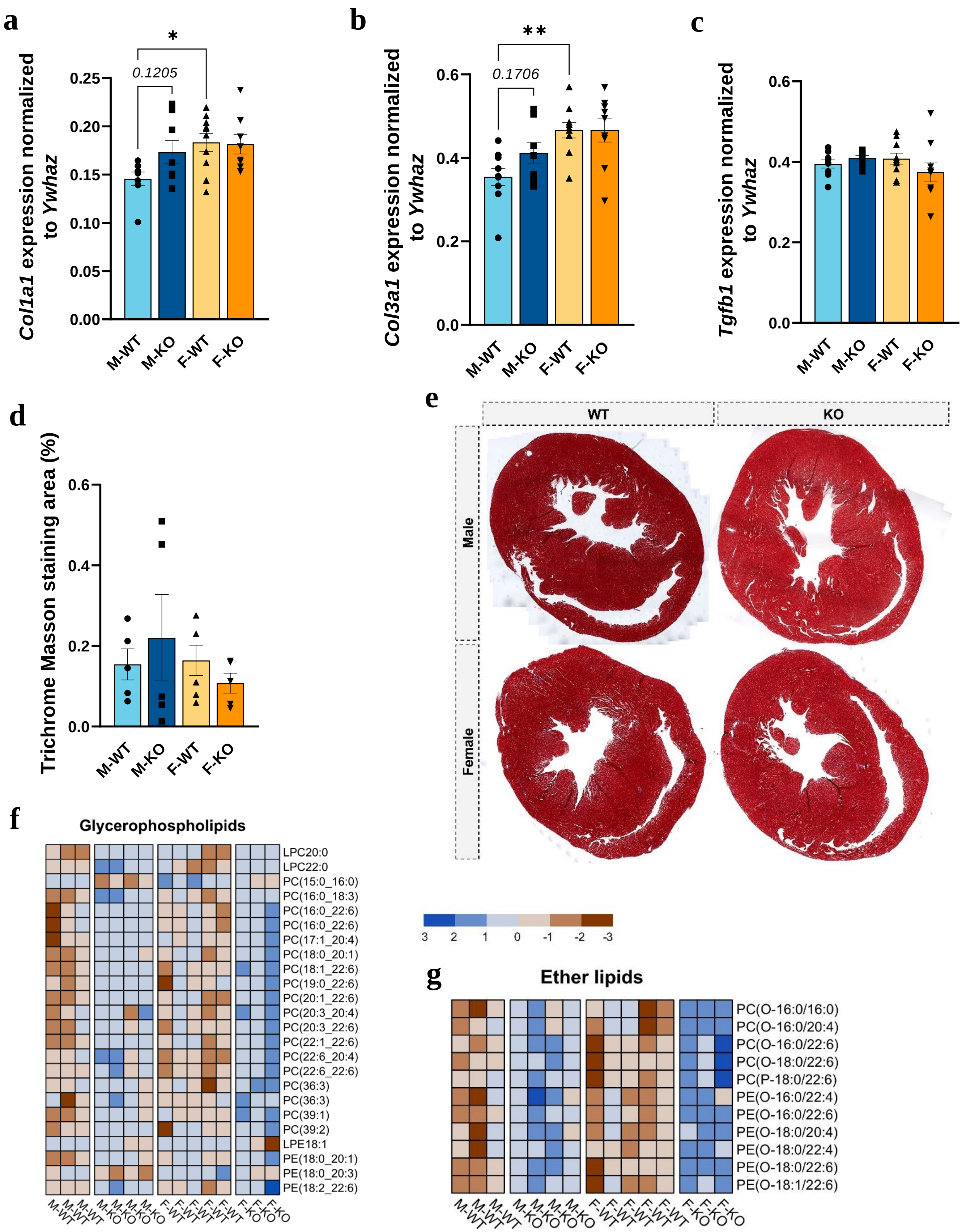
a
b
c
d
e
f
g
Col1a1 expression normalized to Ywhaz
Col3a1 expression normalized to Ywhaz
Tgfb1 expression normalized to Ywhaz
Trichrome Masson staining area (%)
0.1205
0.1706
*
**
M-WT
M-KO
F-WT
F-KO
WT
KO
Male
Female
Glycerophospholipids
Ether lipids
LPC20:0
LPC22:0
PC(15:0_16:0)
PC(16:0_18:3)
PC(16:0_22:6)
PC(16:0_22:6)
PC(17:1_20:4)
PC(18:0_20:1)
PC(18:1_22:6)
PC(19:0_22:6)
PC(20:1_22:6)
PC(20:3_20:4)
PC(20:3_22:6)
PC(22:1_22:6)
PC(22:6_20:4)
PC(22:6_22:6)
PC(36:3)
PC(36:3)
PC(39:1)
PC(39:2)
LPE18:1
PE(18:0_20:1)
PE(18:0_20:3)
PE(18:2_22:6)
PC(O-16:0/16:0)
PC(O-16:0/20:4)
PC(O-16:0/22:6)
PC(O-18:0/22:6)
PC(P-18:0/22:6)
PE(O-16:0/22:4)
PE(O-16:0/22:6)
PE(O-18:0/20:4)
PE(O-18:0/22:4)
PE(O-18:0/22:6)
PE(O-18:1/22:6)
3 2 1 0 -1 -2 -3

Supplementary Figure 3 – Fibrotic and lipidomic phenotype in the heart of WT and *Lrpprc* KO mice. Several cardiac markers were quantified by RT-qPCR (n=9-10): **(a)** *Col1a1* and **(b)** *Col3a1* for fibrosis, and **(c)** *Tgfβ1* for inflammation. **(d)** Masson's trichrome stain area coverage in % (n=8), and **(e)** most representative images. Heatmaps selected lipids significantly discriminating KO from controls mice ($p-corr<0.1), identified by MS/MS using LC-QTOF. Each dot represents a log2-transformed KO/WT signal intensity ratio (n=7-8) for the indicated lipid (sub)classes with their acyl side chain(s) – **(f)** glycerophospholipids, and **(g)** ether lipids. The underscore symbol "_" beside the acyl side chain for PCs, PEs, and TGs refers to acyl chains for which the sn position remains to be ascertained. Statistics: differences between WT and KO mice were assessed with two-way ANOVA tests followed by Šidák multiple comparison post hoc analysis. *p < 0.05, **p < 0.01 before and $p-corr < 0.05 after Benjamini-Hochberg correction. Related to figures 6 and 8.

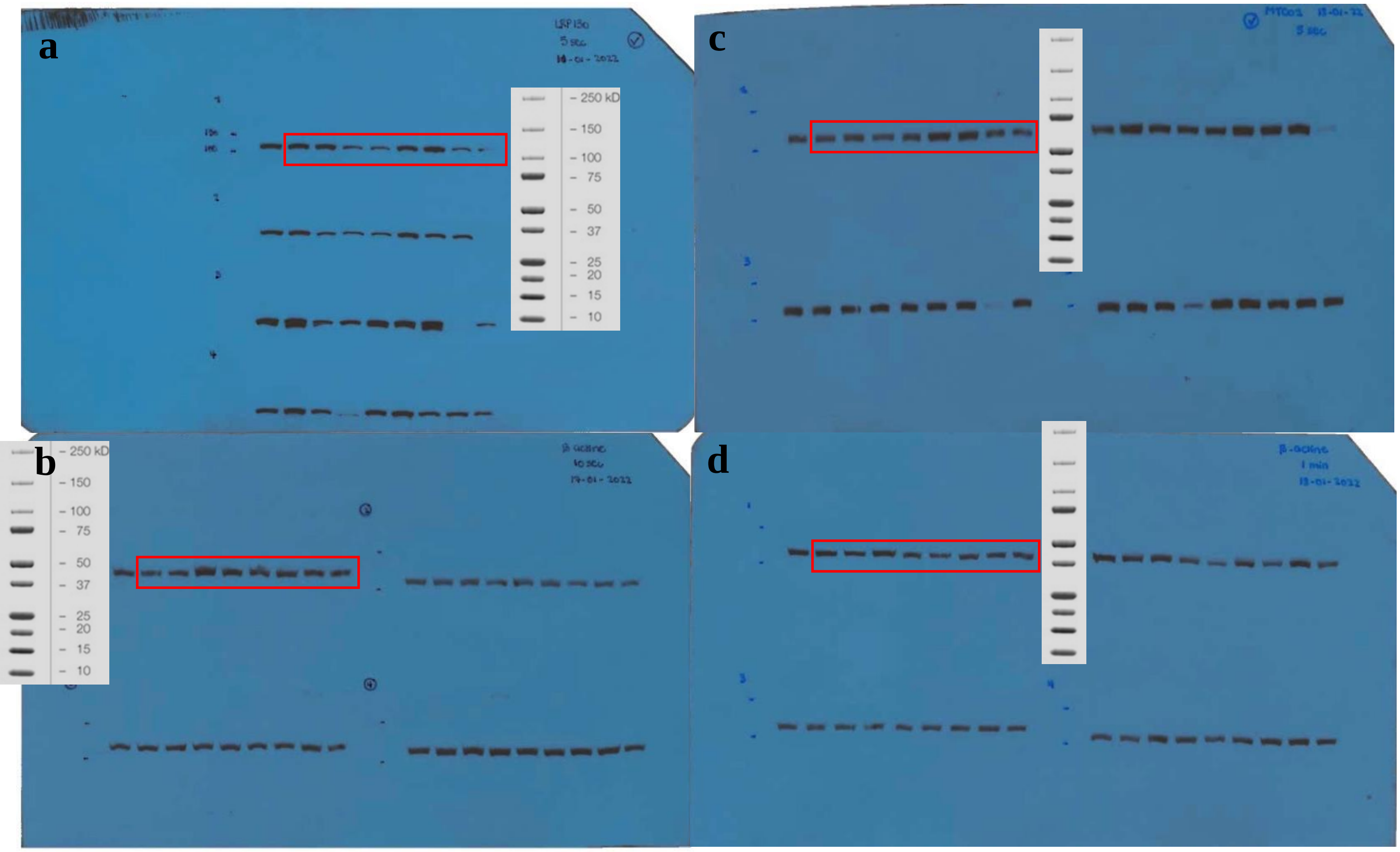

Supplementary Figure 4 – Uncropped liver immunoblots images in normal and liver-specific LRPPRC deficient mice for LRPPRC and MTCO1 protein content from Figure 1a & b. Uncropped and unedited immunoblots images from: **(a)** Figure 1a for hepatic LRPPRC protein content (~130kDa) which was normalized to **(b)** hepatic β-Actin protein content (~42kDa); and from **(c)** Figure 1b for hepatic MTCO1 protein content (~57kDa) which was normalized to **(d)** hepatic β-Actin protein content (~42kDa). Final samples chosen for publication are delimited by a red rectangle and size ladder is aligned with them.

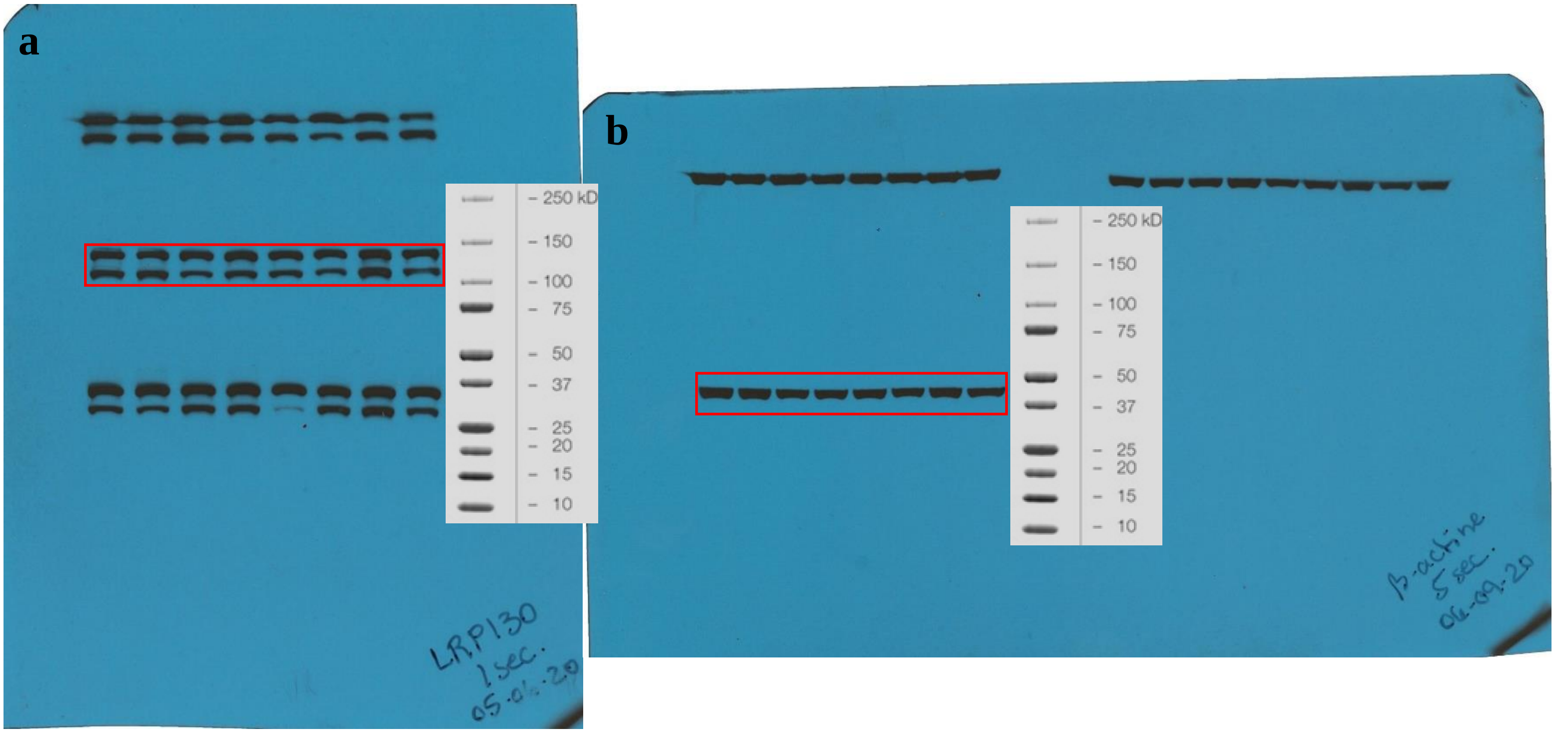

Supplementary Figure 5 – Uncropped heart immunoblots images in normal and liver-specific LRPPRC deficient mice for LRPPRC protein content from Figure 6a. Uncropped and unedited immunoblots images from: **(a)** Figure 6a for cardiac LRPPRC protein content (~130kDa) which was normalized to **(b)** β-Actin protein cardiac content (~42kDa). Final samples chosen for publication are delimited by a red rectangle and size ladder is aligned with them.

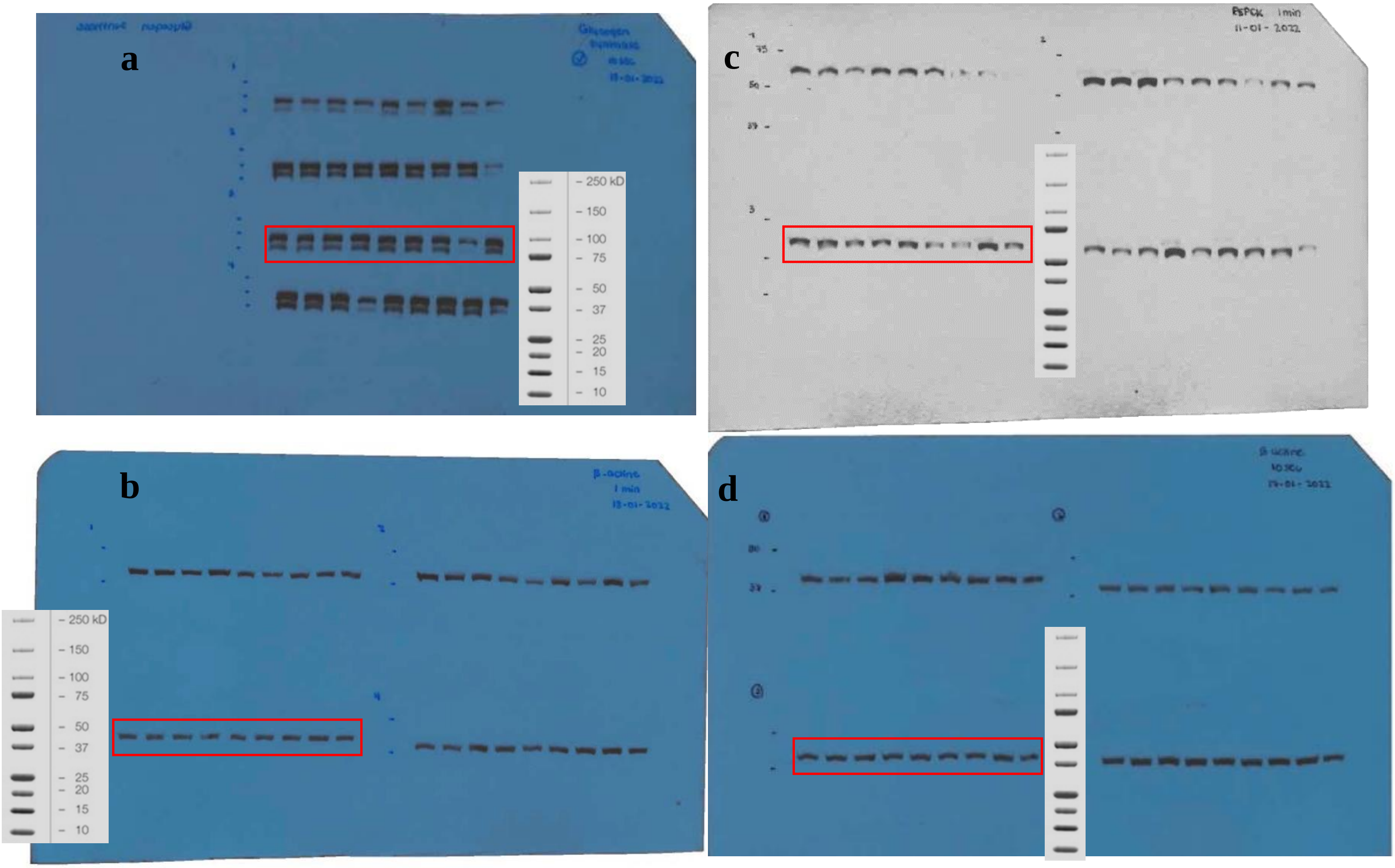

Supplementary Figure 6 – Uncropped liver immunoblots images in normal and liver-specific LRPPRC deficient mice for glycogen synthase (GS) and PEPCK protein content from Supplementary Figure 2. Uncropped and unedited immunoblots images from: **(a)** supplementary figure 8a hepatic glycogen synthase (GS) protein content (~84kDa) which was normalized to **(b)** hepatic β-Actin protein content (~42kDa) and from **(c)** supplementary figure 8b hepatic PEPCK protein content (~63kDa) which was normalized to **(d)** hepatic β-Actin protein content (~42kDa). Final samples chosen for publication are delimited by a red rectangle and size ladder is aligned with them.
