# Supplementary material for: A genetic mouse model of lean-NAFLD unveils sexual dimorphism in the liver-heart axis
Source: Commun Biol. 2024 Mar 22;7:356. doi: 10.1038/s42003-024-06035-6 (PMC10959946; doi:10.1038/s42003-024-06035-6)
Supplement: Supplementary file 2 — Description of Additional Supplementary Files [file 42003_2024_6035_MOESM2_ESM.pdf]

Description of Additional Supplementary Files

File name: Supplementary Data 1
Description: Raw data from transcriptomics related to Figure 4.

File name: Supplementary Data 2
Description: RNA-sequencing in liver of female mice KO vs WT.

File name: Supplementary Data 3
Description: RNA-sequencing in liver of male mice KO vs WT.

File name: Supplementary Data 4
Description: RNA-sequencing in liver of female mice WT vs male mice WT.

File name: Supplementary Data 5
Description: List of lipids identified by MS/MS and significantly discriminating hearts of *Lrpprc* KO from controls mice.
