# Supplementary material for: A genetic mouse model of lean-NAFLD unveils sexual dimorphism in the liver-heart axis
Source: Commun Biol. 2024 Mar 22;7:356. doi: 10.1038/s42003-024-06035-6 (PMC10959946; doi:10.1038/s42003-024-06035-6)
Supplement: Supplementary file 8 — Reporting Summary [file 42003_2024_6035_MOESM8_ESM.pdf]

nature portfolio

Corresponding author(s): Matthieu Ruiz

Last updated by author(s): Feb 26, 2024

Reporting Summary

Nature Portfolio wishes to improve the reproducibility of the work that we publish. This form provides structure for consistency and transparency in reporting. For further information on Nature Portfolio policies, see our Editorial Policies and the Editorial Policy Checklist.

Statistics

For all statistical analyses, confirm that the following items are present in the figure legend, table legend, main text, or Methods section.

n/a	Confirmed	
☐	☒	The exact sample size (*n*) for each experimental group/condition, given as a discrete number and unit of measurement
☐	☒	A statement on whether measurements were taken from distinct samples or whether the same sample was measured repeatedly
☐	☒	The statistical test(s) used AND whether they are one- or two-sided<br>*Only common tests should be described solely by name; describe more complex techniques in the Methods section.*
☒	☐	A description of all covariates tested
☐	☒	A description of any assumptions or corrections, such as tests of normality and adjustment for multiple comparisons
☐	☒	A full description of the statistical parameters including central tendency (e.g. means) or other basic estimates (e.g. regression coefficient) AND variation (e.g. standard deviation) or associated estimates of uncertainty (e.g. confidence intervals)
☐	☒	For null hypothesis testing, the test statistic (e.g. *F*, *t*, *r*) with confidence intervals, effect sizes, degrees of freedom and *P* value noted<br>*Give P values as exact values whenever suitable.*
☒	☐	For Bayesian analysis, information on the choice of priors and Markov chain Monte Carlo settings
☒	☐	For hierarchical and complex designs, identification of the appropriate level for tests and full reporting of outcomes
☒	☐	Estimates of effect sizes (e.g. Cohen's *d*, Pearson's *r*), indicating how they were calculated

Our web collection on statistics for biologists contains articles on many of the points above.

Software and code

Policy information about availability of computer code

Data collection	No software was used in this study.
Data analysis	No software was used in this study.

For manuscripts utilizing custom algorithms or software that are central to the research but not yet described in published literature, software must be made available to editors and reviewers. We strongly encourage code deposition in a community repository (e.g. GitHub). See the Nature Portfolio guidelines for submitting code & software for further information.

Data

Policy information about availability of data

All manuscripts must include a data availability statement. This statement should provide the following information, where applicable:

- Accession codes, unique identifiers, or web links for publicly available datasets
- A description of any restrictions on data availability
- For clinical datasets or third party data, please ensure that the statement adheres to our policy

All data supporting the findings of this study are available within the paper and its Supplementary Information. Uncropped and unedited blot photos with their aligned size markers can be found in the Supplementary Information PDF file, supplementary Figure 11-13. RNA – sequencing data can be found in the Supplementary Data 1 Excel file as well as deposited on NIH’s Sequence Read Archive (SRA), submission code: SUB14265919 and BioProject ID: PRJNA1079766.

Research involving human participants, their data, or biological material

Policy information about studies with human participants or human data. See also policy information about sex, gender (identity/presentation), and sexual orientation and race, ethnicity and racism.

Reporting on sex and gender	This study does not include any human participants, therefore, human sex and gender was not considered.
Reporting on race, ethnicity, or other socially relevant groupings	N/A
Population characteristics	N/A
Recruitment	N/A
Ethics oversight	N/A

Note that full information on the approval of the study protocol must also be provided in the manuscript.

Field-specific reporting

Please select the one below that is the best fit for your research. If you are not sure, read the appropriate sections before making your selection.

☒ Life sciences ☐ Behavioural & social sciences ☐ Ecological, evolutionary & environmental sciences

For a reference copy of the document with all sections, see nature.com/documents/nr-reporting-summary-flat.pdf

Life sciences study design

All studies must disclose on these points even when the disclosure is negative.

Sample size	No sample size calculation. We used a N number according to our previous work.
Data exclusions	No data were excluded.
Replication	N number was chosen accordingly.
Randomization	Randomization is not relevant in this study as mice were specifically selected based on their genotype and sex and thus included in the corresponding group.
Blinding	Blinding was not possible as the genotype and sex of each mouse was known to the investigators. Moreover, generated results were not dependent on or influenced by the investigators' knowledge of the mouse's sex and genotype.

Reporting for specific materials, systems and methods

We require information from authors about some types of materials, experimental systems and methods used in many studies. Here, indicate whether each material, system or method listed is relevant to your study. If you are not sure if a list item applies to your research, read the appropriate section before selecting a response.

Materials & experimental systems

n/a	Involved in the study	
☐	☒	Antibodies
☒	☐	Eukaryotic cell lines
☒	☐	Palaeontology and archaeology
☐	☒	Animals and other organisms
☒	☐	Clinical data
☒	☐	Dual use research of concern
☒	☐	Plants

Methods

n/a	Involved in the study	
☒	☐	ChIP-seq
☒	☐	Flow cytometry
☒	☐	MRI-based neuroimaging

Antibodies

Antibodies used	LRP130 Rabbit Ab cat. PA5-22034, Anti-MTCO1 antibody [1D6E1A8] cat. ab14705, Glycogen Synthase (15B1) Rabbit mAb cat.3886S, PEPCK (H-300) Rabbit polyclonal IgG cat. sc-32879, β-actin HRP (C4) cat. sc-47778, Recombinant Anti-F4/80 antibody [EPR26545-166]

	(ab300421), Goat Anti-Rabbit IgG H&L (HRP) (ab205718).
Validation	Validated by the manufacturer.

Animals and other research organisms

Policy information about studies involving animals; ARRIVE guidelines recommended for reporting animal research, and Sex and Gender in Research

Laboratory animals	Mouse, male & female, study conducted when mice were aged from 8 to 14 weeks.
Wild animals	Mouse, male & female, study conducted when mice were aged from 8 to 14 weeks.
Reporting on sex	This study was performed on male and female mice. Sex related differences were indeed reported and described as such. The male and female sex in this study are clearly identified throughout the manuscript as well as in all the figures.
Field-collected samples	Mice were maintained in a specific pathogen-free facility on a regular 12-12h light-dark cycle. All mice had access to standard chow diet and water ad libidium. Plasma was recovered following centrifugation (2000g, 15min, 4°C), and stored at -80°C pending further experimentations. The heart, liver, perigonadal white adipose tissue (WAT), interscapular brown adipose tissue (BAT), gastrocnemius, soleus, and tibia were collected, rinsed in cold 0.9% saline, weighted, flash frozen in liquid nitrogen, and stored at -80°C.
Ethics oversight	The study protocol was approved by the animal research and ethics committee of the Montreal Heart Institute.

Note that full information on the approval of the study protocol must also be provided in the manuscript.

Plants

Seed stocks	Our study does not involve plants.
Novel plant genotypes	Our study does not involve plants.
Authentication	Our study does not involve plants.
